# Coatings Based on Refractory Materials for Corrosion and Wear Applications

**DOI:** 10.3390/ma17235936

**Published:** 2024-12-04

**Authors:** Natalia A. Shapagina, Vladimir V. Dushik

**Affiliations:** Frumkin Institute of Physical Chemistry and Electrochemistry Russian Academy of Sciences, Leninsky Prospect 31-4, 119071 Moscow, Russia; v.dushik@gmail.com

**Keywords:** refractory metals, coatings based on refractory metals, nanostructured and microstructured coatings, methods of synthesizing refractory coatings, corrosion, physical mechanics, tribology

## Abstract

Coatings based on refractory metals and compounds have been used in various industries since the last century due to their high thermal and heat resistance, as well as their excellent mechanical and tribological properties. Advances have made it possible to apply high-tech methods for their production, which has improved their availability and expanded their range of applications. A promising area of use of coatings based on refractory systems is the anticorrosion protection of structural materials. The high wear resistance and anticorrosion ability of these materials will allow for the protection of critical units of equipment of various industries from the complex destructive effects of factors of chemical and mechanical nature. For the effective choice of coating composition, it is necessary to know the basic characteristics of refractory material layers and the method of their production. The purpose of this article is to summarize modern scientific data on methods of obtaining refractory coatings, as well as on their composition, structure, and protective properties. The information presented in this review will bridge the gap between research and industrial development and expand the niche area of utilization.

## 1. Introduction

Corrosion—the spontaneous degradation of metals and alloys due to their chemical or electrochemical interaction with the environment—is one of the dominant factors in the loss of metal stock in the world [1]. In view of the wide spread of metallic materials in the world economy, in particular alloys of iron, copper, nickel, aluminum, zinc, and titanium, the issue of protection against corrosion does not lose its relevance. Thanks to the success of corrosion science, the basic mechanism of the electrochemical corrosion of metals is now well known, and the basic principles of corrosion protection have been formulated. These include electrochemical and inhibitor protection, anticorrosion coating, and corrosion-resistant alloying. Modern industry, armed with the achievements of corrosion science, creates effective corrosion-resistant alloys for a wide range of applications based on the available metals—iron, aluminum, copper, nickel, and chromium. However, the search for new materials has not stopped, and metals and alloys that were not even considered as anticorrosive before are being used. In this connection, refractory metals and their coatings attract special attention.

Refractory metals are a group of metals and alloys that have a high melting point, usually above 2000 °C. Refractory metals include 13 elements of the periodic system: tungsten, molybdenum, niobium, tantalum, vanadium, hafnium, chromium, rhenium, technetium, ruthenium, rhodium, osmium, and irridium. Due to their unique properties, they are widely used in various fields of modern technology, from rocket construction to electronics. Most refractory metals are intensively oxidized to form volatile oxides when heated in air to temperatures above 600 °C. Therefore, as heating elements, they work in a vacuum or in a protective inert medium, e.g., argon [2,3].

According to the definition, a nanomaterial is a material with at least one of its dimensions at the nanometer scale, i.e., <100 nm, in three-dimensional space [4]. Due to their miniaturized dimensions, nanomaterials have a high ratio of surface area to volume, which makes it possible to impart the necessary physical and chemical properties that micro- and macroscale materials do not have. Nanomaterials have found applications in various fields, such as the optical, thermal, mechanical, chemical, magnetic, medical, electrical electronics, and energy storage fields [4].

As mentioned earlier, corrosion processes have a negative impact on the service life of various metal objects and structures, which stimulates the development of anticorrosion methods to counteract them. One of the proven means of protection is the application of coatings to critical parts, structures, and facilities. Understanding the physical and mechanical properties of coating/substrate systems is the basis for successful corrosion and wear protection, as coatings are subjected to high mechanical loads, thermal stresses, and temperature effects over a wide range (from 100 to 2700 °C) [5].

According to various literature data [6,7], the use of nanostructured coatings provides improved surface protection. However, there is still an unresolved question: does the presence of nanostructures in refractory metal coatings allow them to resist corrosion damage or is the anticorrosion effect achieved by the presence of alloying additives? This review includes (i) information on refractory metals; (ii) a description of methods for synthesizing coatings based on refractory materials; and (iii) information on the corrosion resistance of refractory coatings obtained by different synthesis methods.

## 2. Refractory Metals and Compounds

### 2.1. Thirteen Refractory Metals of the Periodic System

The refractory metals to which this review is devoted are summarized in Table 1.

As can be seen from Table 1, refractory metals are used in almost all spheres of human activity.

### 2.2. Methods of Deposition of Refractory Coatings

Among the modern industrial methods of the formation of refractory micro- and nanostructured coatings on structural materials to protect the surface from abrasive and corrosive wear, the following can be distinguished:

- Chemical vapor deposition (CVD);
- Physical vapor deposition (PVD);
- Spray coating;
- Composite electroplating;
- Chemical heat treatment (CHT);
- Self-propagating high-temperature synthesis (SHS);
- Powder cladding;
- Electrospark alloying.

Basic information on each method is presented below.

#### 2.2.1. CVD Method

**Figure 1:**
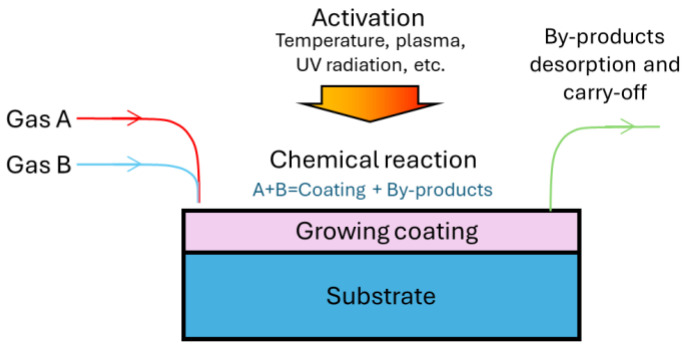
CVD process diagram.

The essence of the CVD method is the deposition of solid layers in specially prepared atmospheres as a result of the chemical conversion of gas mixture components on the coated surface. A generalized scheme of the CVD processes is presented in Figure 1.

The process of solid product layer deposition from the vapor–gas phase (CVD kinetics) depends on a number of parameters: the temperature, the pressure, the concentration of reagents in the gas mixture, the ratio of these reagents, the gas flow rate, the nature of substances, and others. To intensify these processes, additional energy sources can be used, such as plasma, ultraviolet, and laser radiation [8,26].

Obviously, the CVD process has a number of limitations, e.g., it depends on the type of possible reactions and the minimum substrate temperature to be maintained. These circumstances impose limitations on the possible configuration of the substrate/coating system and on the nature of the process control. The existing industrial methods allow for the expansion of the set of product combinations, to significantly reduce the process temperature, to practically eliminate the mismatch in morphological parameters during nucleation and growth, to change the physicochemical properties in the desired direction, etc. With the help of this technology, it is possible to obtain coatings from virtually any suitable for these purposes metals and nonmetals, including carbon and silicon, as well as compounds (carbides, nitrides, borides, oxides, intermetallides, etc.). This is the most technologically advanced method in modern industry, which meets the requirements of “flexible” technology. The main advantage of the CVD method is that the coating process uses gaseous media, which makes it possible to apply coatings in hard-to-reach places, such as small-diameter holes, the internal cavities of pipes and vessels, and so on. The coating material fills the smallest pores in the surface [26].

Vapor deposition processes are classified by the operating pressure, type of activation effect, type of working substance, physical characteristics of vapor, and other features of the process. The most common classification differentiates CVD processes by the pressure in the reactor [27] as follows:

- Atmospheric-Pressure Chemical Vapor Deposition (APCVD) and Subatmospheric (Pressure) Chemical Vapor Deposition (SACVD)—the process takes place at atmospheric and subatmospheric pressure, and the equipment is simple but easily leads to particle contamination.

- Low-Pressure Chemical Vapor Deposition (LPCVD)—the process is carried out in a vacuum at pressures below atmospheric pressure, usually below 10^2^ Pa. The lower pressure reduces the probability of undesirable reactions in the gas phase and leads to a more uniform deposition of the film on the substrate.

- Ultra-High-Vacuum-Pressure Chemical Vapor Deposition (UHVPCVD)—the process is carried out under an ultra-high vacuum, usually more than 10^−6^ Pa. This method is suitable for obtaining films of high purity and quality.

- Atomic Layer Deposition (ALD)—advanced vacuum CVD process, in which reagents are applied to the surface by the gas adsorption effect by short pumping through the working chamber, and the reaction takes place due to short heating at a reactor pressure below 10^2^ Pa, which allows ultrathin coatings to form.

Most modern pressure CVD plants use either LPCVD or UHVPCVD, in which various types of reactions such as pyrolysis, redox reactions, hydrolysis, and complex compound formation take place.

As mentioned above, depending on the method of decomposition of the feed gases used in the CVD process, additional energy sources may be utilized. In this case, CVD processes are categorized into the following:

- TCVD (Thermal–Chemical Vapor Deposition)—the process takes place at high-temperature with the formation of thin films due to a chemical reaction between the components of the gas mixture;

- PECVD (Plasma-Enhanced Chemical Vapor Deposition)—the chemical process of coating deposition is activated by plasma, which allows the process temperature to be significantly reduced;

- Laser–CVD (Laser Chemical Vapor Deposition)—the process uses a laser beam to initiate or stimulate a chemical reaction, allowing for localized fine deposition;

- PACVD (Photo-Assisted Chemical Vapor Deposition)—a light energy source (usually ultraviolet radiation) is used to stimulate chemical reactions in the process.

#### 2.2.2. PVD Method

**Figure 2:**
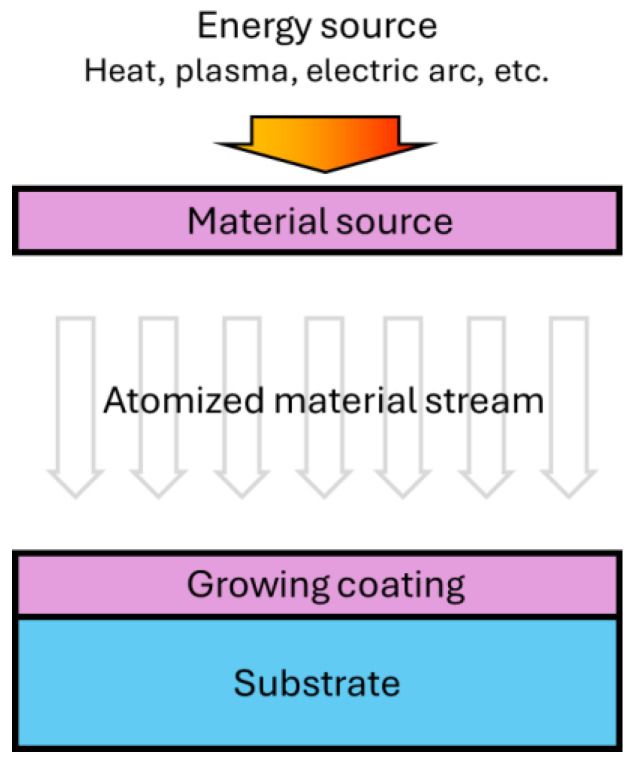
PVD process diagram.

The essence of the PVD process involves the creation of a directed flow of sputtering particles (atoms, molecules, or clusters) in a vacuum environment in the form of vapor or plasma and its subsequent deposition on the surface to be treated (Figure 2). The PVD process should be performed in a high-vacuum or low-pressure environment to allow the free propagation of material vapor or ions and avoid collisions with gas molecules, in order to avoid reducing the deposition efficiency. Vacuum pumps of different designs are used for the evacuation of working chambers. By controlling the deposition conditions, such as the gas flow, offset voltage, vaporization energy, and composition of targets, the precise control of film thickness, composition, and properties can be achieved to meet the needs of various applications [8,27,28].

The main stages of the PVD process are as follows [28]:

- Change of the condensed phase of the substance into the gaseous phase—at this stage, the material is heated to the temperature of vaporization or atomized under the influence of plasma. When heated or atomized, the surface atoms or molecules of the material receive enough energy to overcome the surface tension and change to a gaseous state.

- Creation of a flow of substance particles towards the object surface, which is achieved either by simple diffusion in a vacuum medium or by creating an electrostatic field.

- Particle settling on the surface which forms a continuous film increasing in thickness.

Thus, a thin-film coating is formed, the thickness and properties of which can be adjusted by controlling the process parameters.

There are several techniques for the PVD process [27,28]:

- Magnetron sputtering—in this case, magnetic fields are used to manipulate ions and bombard solid targets and generate high-energy ions and release them onto the target surface. The peculiarity of this technique is the higher deposition rate and more uniform film thickness distribution. Magnetron sputtering is commonly used in the manufacture of semiconductor devices, optical films, etc.

- Electron beam evaporation—electron beams are used to heat materials and vaporize them to form films. This method takes place at a higher temperature and has a high deposition rate, which is suitable for the deposition of materials with a high melting point. Electron beam evaporation is mainly used to prepare metallic coatings and films or other materials with a high melting point.

- Laser ablation—a laser is used to heat the material, causing its vaporization and deposition on the substrate surface. The use of this technique allows for the localized deposition of the film. Laser ablation is used to obtain functional coatings, nanostructures, etc.

- Thermal evaporation—in this situation, the material is heated to the vapor evaporation temperature. This technique is used to prepare metal films or other low-melting-point materials for deposition. Thermal evaporation is used to prepare metal coatings, optical films, etc.

- Electric arc deposition allows for the achievement of isothermal states in the plasma, which is suitable for creating films of stoichiometric compositions with high adhesion strength.

Thus, each PVD process technique has its own unique principles, characteristics, and applications. The selection of the appropriate technique depends on the specific application requirements, material properties, and expected film characteristics.

#### 2.2.3. Spray Coating Method

**Figure 3:**
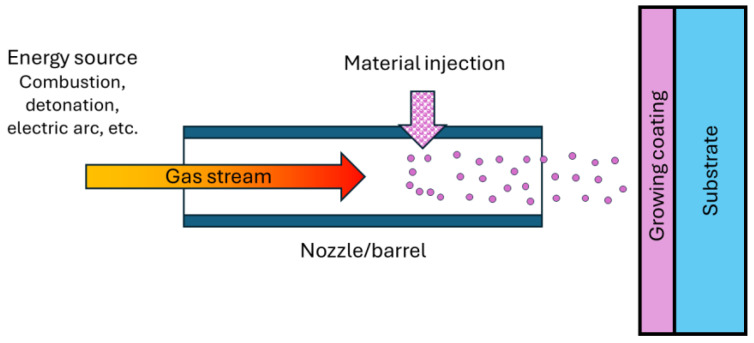
Generalized scheme of spray techniques.

Spraying is a technique in which a target material is passed through a nozzle and deposited on a substrate surface under impact to produce a coating. Either powders or pro-wires (rods) are used to create the particle stream. When using wire, the particle stream is created by melting the wire and atomizing it with a high-speed flow of autonomous gas or by the heat source itself (plasma, gas-flame jet) (Figure 3) [8,29].

Among other coating methods, sputtering technologies have a notable advantage—the versatility of coating material selection (metallic, polymer, ceramic, composite, low- and high-melting-point coatings, amorphous coatings). All spraying technologies can be divided into two groups: gas–thermal spraying and vacuum condensation spraying. Within each group, there are a number of sputtering methods, differing in the source of the supplied energy and the physics of the processes. The structure of the coating material is formed by the impact, deformation, and solidification of heated particles on the surface of the base (substrate) or previous cooled particles. This forms a layered material consisting of deformed particles (splats) connected by contact areas. Depending on the heat source and driving forces of transfer, the following sputtering methods are distinguished: plasma, gas-flame, detonation, arc metallization, and high-frequency metallization. In the first three methods, the heating source is combined with a source of atomization and particle acceleration. In arc and high-frequency metallization, the heating and acceleration sources are separated [8,30].

##### Thermal Spraying

The technology of the thermal spraying of coatings is a complex set of physical and chemical mechanical phenomena. It can be divided into two main stages: the formation of a two-phase flow (gas jet with dispersed particles) and the formation of the coating itself. At the first stage, an interaction between the high-temperature gas stream and particles occurs, including the heat exchange and heating of particles and their acceleration by the transfer of kinetic energy from the gas stream jet. In the second step, a coating is formed when the molten particles collide with the surface of the substrate, causing the particles to lie tightly on top of each other and, spreading out, fill in the irregularities of the rough surface. With the help of gas–thermal spraying technology, it is possible to obtain coatings with a specified hardness, wear resistance, heat resistance, antifriction property, and corrosion resistance. The high efficiency and versatility of this method is determined by the following features: the possibility of coating various materials—pure metals, alloys, oxides, borides, carbides, and their combination in various proportions; the possibility of obtaining coatings on a variety of structures—on metals, glass, ceramics, plastics, etc.; the small deformation of the sprayed product; the low consumption of coating material, the thickness of which is 0.1–10 mm; and high productivity. As it was mentioned earlier, gas–thermal spraying is subdivided into the following [30]:

- Plasma spraying—this method allows for coatings that meet the above requirements to be obtained because it is possible to control the process energy within wide limits (the operating temperature of the plasma jet varies from 5000 to 15,000 K) and to create coatings of almost any material, including composite materials that cannot be obtained by other methods. The disadvantages of the method include the relatively low productivity of the sputtering process, noise during operation, intensive ultraviolet radiation, and relatively low adhesion strength and density caused by the low kinetic energy of the spray [31,32].

- Detonation spraying—this method uses a specific method of heating, atomization, and acceleration of the sprayed particles. The source is a high-velocity flow of a gas mixture produced by a directed detonation explosion. Detonation is the process of the chemical transformation of an explosive when a detonation wave propagates through it at the highest possible velocity exceeding the speed of sound in a given medium. The detonation wave is formed in the chamber of the water-cooled barrel of the unit (detonation gun), into which the powder of the material to be sprayed is fed simultaneously with the working gas mixture. The velocity of detonation products determines the velocity of the sprayed particles (800 ÷ 1200 m/s). The high speed of the particles’ movement and their heating during detonation sputtering ensures that high-density coatings with a high adhesion strength to the substrate are obtained. At the same time, the temperature of the base material remains low, excluding its deformation or other physical change, which allows for the use of this method of spraying for precision parts and to obtain coatings with high adhesion. The disadvantages of the method include noise (up to 140 dB), because of which the equipment is installed in a chamber with soundproof walls, as well as its low productivity [30,33].

- Gas dynamic spraying—since as a result of spraying the material is heated to the melting temperature (and even higher) and then with the help of a high-temperature jet is delivered to the sprayed surface, it is necessary to take into account that during all these actions there are complex physical and chemical processes, including oxidation reactions, the burnout of finely dispersed powders, and the decomposition of a number of materials. Thus, along with positive processes, there are also negative ones associated with the use of high-temperature gas or plasma flows. To date, approaches have been proposed and implemented to reduce the impact of negative factors on the physical and technical characteristics of coatings associated with the optimization of gas–thermal spraying processes. The technology of “cold” gas dynamic spraying allows for this important problem to be solved in a fundamentally different way, using not thermal energy but the kinetic energy of the sprayed particles accelerated by cold gas as the main one and thus leveling the negative high-temperature effects. The essence of this technology is that the coatings are formed from non-melted high-speed particles of 50 ÷ 0.01 microns in size, accelerated in supersonic aerodynamic installations to a speed of 1000 m/s and more at the temperature of the working gas significantly below the melting point of the particle material. Thus, the distinctive feature of the technology of “cold” gas dynamic spraying (in comparison with plasma, gas–plasma, detonation) is that the main energy source of coating formation is the kinetic energy of the sprayed particles, imparted to them by the supersonic working gas flow, which provides coatings with minimum temperature stresses without through-pores and microcracks and determines high conductive, anticorrosive, and strength (adhesion and cohesion) properties [30,34].

##### Vacuum Condensation Spraying

In this technology, the coating is formed from streams of particles in atomic, molecular, or ionized states. The process of coating formation can be divided into three stages: the transition of the condensed phase into the gaseous phase; the formation of the flow and transfer of particles to the surface of the product; and vapor condensation on the spraying surface and coating formation. To obtain coatings with high quality, it is required to strictly observe the optimal modes of processes at each of the three stages. The advantages of vacuum condensation sputtering include the good physical and mechanical properties of coatings; the possibility of obtaining coatings from synthesized compounds (carbides, oxides, nitrides, etc.); and obtaining thin and uniform coatings (from tens of nanometers to hundreds of micrometers from various materials). The disadvantages of this sputtering technology include the low productivity of the process (sputtering speed of about 1 micrometer/minute); increased complexity of the technology and equipment (associated with the vacuum system); and low energy coefficients of sputtering [8,30].

Various sources of energy impact on the material are used to produce a particle stream (vapor). A distinction is made between particle flux generation by the thermal vaporization of the material, ion sputtering, or explosive vaporization–sputtering. Accordingly, vacuum condensation spraying is divided into the following methods [30]:

- Thermal evaporation or thermal vacuum spraying—in this case, various sources of thermal energy are used to heat the material and its subsequent evaporation: resistive heaters, electronic or light (laser) beams, induction heating, or arc heating. To improve the quality of the coating, it is recommended to heat the surface or increase the speed of sprayed particles. To increase the particle energy, various methods of influencing the particle flow are used [35,36,37].

- Explosive atomization—this process takes place at the local impact of the energy pulse on the surface of the material, as a result of which conditions for the high-speed vaporization of solid materials are created on microareas of the surface. The atomization–evaporation process takes place with the formation of a stream of sprayed particles. The main part of the atomization products consists of the vapor phase. However, almost always, the atomization–evaporation process is accompanied by the formation of tiny particles of the condensed phase. The size of the particles forming the droplet phase ranges from fractions to tens of microns. The droplet fraction getting into the coating reduces the quality of the latter, leading to the inhomogeneity of the micro- and macrostructure. The condition of explosive atomization–evaporation can be obtained with the help of various pulse sources of thermal energy moving on the surface of the atomized material. For this purpose, electron or laser beams or electric discharges are used. The process of the atomization of a material when a negative potential of a power source is applied to it has found wide application. In practice, such a discharge is called a reverse polarity arc or cathodic form of arc. Arc discharge exists in the vapors of atomized cathode material. The high current density in cathode spots provides a mode of explosive vaporization. This method of vacuum coating production is called the ion–plasma vacuum spraying method because the formation of coatings in this case occurs mainly from ionized particle flow. The special features of the method of explosive spraying include the versatility of the sprayed materials; high productivity; high adhesion; and uniform coating thickness. The disadvantages of the method include the presence of a droplet fraction, which forces the sprayed stream to separate [30,38].

- Ion sputtering—when using this method, the material to be sprayed is bombarded with an accelerated stream of positively charged particles (argon ions and other inert gases). The atomized material acts as a cathode. The atomization of the cathode occurs as a result of the direct transfer of the positive ion impulse to the surface atoms, provided that the ion energy exceeds a certain threshold value (the energy of atom lattice bonding). Hence, another name of the method is cathodic sputtering. The advantages of the method include obtaining coatings from alloys practically without changing their composition in the process of sputtering and a high material utilization factor approaching unity. The disadvantages of the method include the low energy efficiency of the process and the weak degree of ionization of the sprayed stream [39].

- Vacuum condensation reaction sputtering—a chemically active gas is injected into the chamber during sputtering, resulting in a reaction between the vapor flow of the atomized metal and the atoms of the specially introduced gas. Solid reaction products are deposited on the sputtering surface. This method is useful for obtaining coatings from certain carbides, oxides, nitrides, and other compounds that undergo partial or complete decomposition when heated and atomized by conventional methods. Reaction sputtering can be carried out on the basis of any of the vacuum condensation sputtering methods discussed above. The higher gas pressure in the chamber during reaction sputtering compared to the direct evaporation or atomization of the material increases the collision and diffusion scattering frequency of the condensed atoms. As a result, the coating is deposited uniformly over the entire surface of the product, even if it has a complex shape [40].

#### 2.2.4. Composite Electroplating Coatings

##### Electrochemical Deposition of Coatings

Galvanic coatings have found wide application in industry due to a wide choice of coating material (practically all metals) and the high manufacturability of the process. The technology of electrochemical deposition of coatings can be divided into the following stages [8,30,41]:

- Surface preparation: the substrate material is degreased, then etched, and then activated and placed in a galvanic bath with a selected electrolyte solution;

- An anode and cathode (coated product) are connected in a galvanic bath, and then an electric current is started from an electric source;

- Under the influence of an electric field, particles and metal ions migrate to the surface of the substrate and are deposited on it through the electrochemical reduction reaction;

- After the completion of electrochemical deposition, the process of electric current supply is stopped, the substrate is removed, washed and dried, and, if necessary, additionally treated.

**Figure 4:**
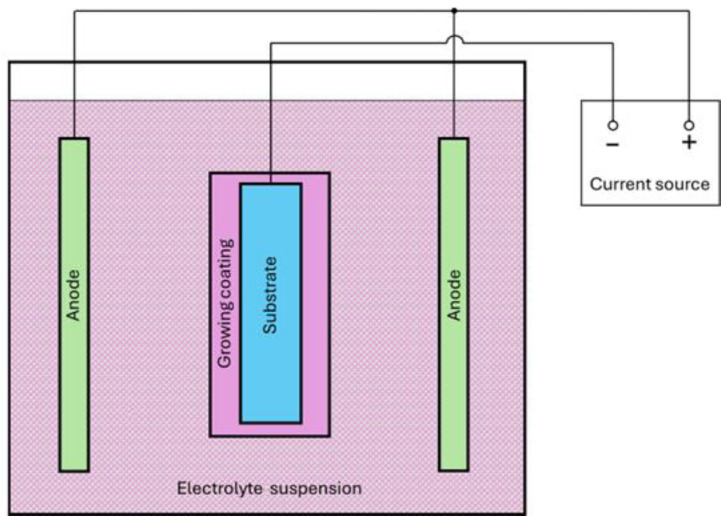
Generalized scheme of electrochemical deposition.

The process is schematically shown in Figure 4.

The electrodeposition process in the galvanic bath is controlled by the following phenomena: In the electrolyte volume, this involves the dissolution of the anode metal, metal precipitation, electrolytic dissociation in the electrolyte, and the movement of reaction products (convection, diffusion, transfer by electric field). At the interface, this involves adsorption on the surface, surface diffusion, and crystallization. In the volume of the product (cathode), this involves volume diffusion. The characteristics of the process and the resulting coating during electrolysis are determined by the following technological parameters: the base material, the coating material and its electrochemical characteristics, the electrolyte composition, the electric current density, the duration and temperature of the process, and the shape of the cathode and its position relative to the anode. These parameters determine the growth rate of the coating, its thickness, the homogeneity of the coating material, the type of surface (smooth, matte), and the porosity of the coating [41].

The electrochemical deposition of alloys provides an opportunity to improve the quality of the deposited coating layers. At normal temperatures and specific conditions determined by crystallization conditions, it is possible to control the composition and structure of the coating and obtain properties that cannot be obtained by other methods. It is also possible to form metastable phases, which at a given chemical composition deviate from the equilibrium diagram for the alloys of a given system. Of particular interest is the possibility to precipitate as components of alloys metals such as tungsten, rhenium, molybdenum, titanium, etc. It should be taken into account that the introduction of such metals in many cases is carried out not in metallic but in oxide or hydroxide form. The important properties of coatings in the form of alloys are magnetic permeability, residual magnetization, superconductivity, brazing ability, wear resistance, and corrosion resistance. In combination with subsequent heat treatment, high values can be obtained in this way [30,41].

##### Metal Matrix Composite Electroplating Coatings

High-performance characteristics of electroplating coatings and their wide application in mechanical engineering were obtained as a result of the development of the technology for obtaining electroplated metal matrix composites. Such coatings are produced from suspensions, which are electrolytes with the addition of a certain amount of highly dispersed powder. The part to be hardened is used as a cathode. When current flows through the electrolyte, metal (first phase—matrix) and powder particles (second phase) are deposited on the part, which are bonded by the matrix material. The presence of the second phase in these coatings significantly affects their physical and mechanical, in particular tribotechnical, properties. The following can be used as second-phase materials: borides, carbides, nitrides, silicides, abrasive powders, diamond, etc. The matrix material in the composite coatings is most often nickel because it has an affinity for most of the particles used as the second phase and easily forms coatings with them. In addition to nickel, iron, cobalt, chromium, copper, and noble metals are also used [30,41].

An important advantage of composite electrochemical coatings compared to galvanic coatings is hardness and strength, which can reach wide limits due to the volume content of the second phase, its size, and the distance between hardening particles. The matrix material has a significant influence on hardness and toughness. The wear resistance of composite coatings is several (sometimes tens) times higher than that of pure coatings. The optimal size and concentration of solid particles in the coating produces favorable conditions for the creation of wear-resistant secondary structures on the friction surface. The plastic deformable matrix evenly redistributes the load over the entire friction area of the part, taking this load from solid inclusions, which are securely held in the matrix. The advantages of composite coatings include the wide choice of initial materials; high quality of the coating (high density, homogeneity, chemical purity); and low temperature of the process (the structure and phase composition of the base material are preserved). The disadvantages include a small coating thickness; a low rate of coating thickness; and the presence of toxic substances in the composition of electrolytes [41].

#### 2.2.5. Chemical Heat Treatment (CHT)

CHT is the process of the surface saturation of the metal with various elements: the process of changing the chemical composition, microstructure, and properties of the surface layer of the part. The change in the chemical composition of surface layers is achieved because of their interaction with the environment (solid, liquid, gaseous, plasma) in which heating is carried out. As a result of changing the chemical composition of the surface layer, its phase composition and microstructure change. The main parameters of chemical–thermal treatment are the heating temperature and holding time. The CHT of metals and alloys is based on the diffusion process. Diffusion is the process of moving atoms or molecules from an area with a higher concentration to an area with a lower concentration [8,42,43].

CHT processes consist of three stages [43]:

- Dissociation consists of the disintegration of molecules and the formation of active atoms of the diffusing element;

- Adsorption—the contact of atoms of the diffusing element with the surface of the steel product and the formation of chemical bonds with metal atoms;

- Diffusion—the penetration of the saturating element into the depth of the metal.

**Figure 5:**
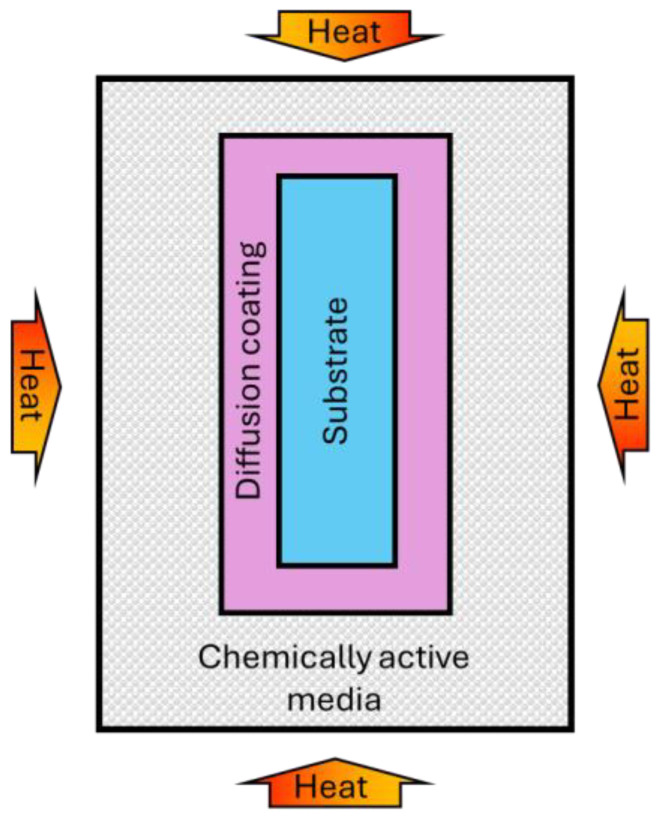
Generalized scheme of CHT.

Various methods of chemical heat treatment are used in industry, differing in the diffusing elements, the type and composition of the external medium, the chemistry of processes, the technique of execution, and other features. Depending on the aggregate state of the external environment in which the treated product is placed, chemical–thermal treatment in solid, liquid, and gas media is distinguished. The generalized scheme of the CHT processes is shown in Figure 5.

The main varieties of chemical heat treatment are as follows [8,43]:

- Cementation—the saturation of the surface layer with carbon, resulting in a hard surface layer that increases the wear resistance of the metal. Cementation is commonly used in the manufacture of gears, bearings, and other components requiring high wear resistance.

- Nitriding—the saturation of the surface layer with nitrogen, resulting in the increased fatigue strength and wear resistance of the metal. Nitriding is commonly used in the production of aerospace components such as turbine blades, landing gear parts, etc.

- Nitrocarburizing and carbonitriding—the saturation of the surface layer with carbon and nitrogen simultaneously, which improves the hardness, wear resistance, and corrosion resistance of the metal. Carbonitriding is commonly used in the manufacture of gears, camshafts, and other components requiring high wear and fatigue resistance. The difference from nitrocarburizing is that carbonitriding introduces a higher level of carbon into the surface layer of the metal, which can give improved performance in terms of wear resistance and thermal resistance. A distinctive feature of nitrocarburizing is that nitrogen and carbon saturation occur simultaneously, which gives higher strength and resistance characteristics to the surface layer.

- Diffusion metallization—the saturation of the surface layer with various metallics and nonmetals such as Al, Cr, V, B, etc. Such types of chemical heat treatments are used to increase the wear, heat, and corrosion resistance, the high-temperature strength of metal and its machinability, etc.

The process of chemical heat treatment can be divided into the following general operations [42,43]:

- Cleaning—the metal surface is thoroughly cleaned of any impurities or contaminants that may interfere with the chemical heat treatment process;

- Preheating—the metal is preheated to a certain temperature to prepare it for the chemical–thermal treatment process;

- Treatment—the metal is placed in a chemical-rich environment for a certain period of time to allow a chemical reaction to take place;

- Cooling—the metal is gradually cooled to room temperature to prevent warping or deformation;

- Post-treatment—depending on the type of chemical heat treatment used, the metal may require additional post-treatment steps, such as polishing or grinding, to achieve the desired surface finish.

Chemical heat treatment can be applied to a wide range of metals, including steel, alumina, titanium, and copper alloys, and can be used in a variety of industries including automotive, aerospace, and manufacturing. To achieve the best results, it is important to select the appropriate chemical heat treatment method based on the desired properties and characteristics of the metal. It is also very important to follow proper chemical heat treatment operations to avoid any problems with warping, deformation, or uneven processing. When the appropriate treatment method is selected and proper procedures are followed, the results can be durable and cost-effective [43].

#### 2.2.6. Formation of Coatings by SHS Method

**Figure 6:**
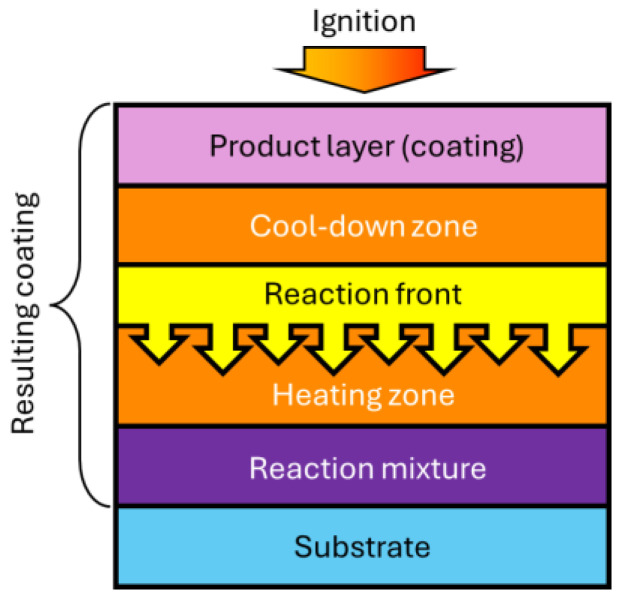
Generalized scheme of SHS.

The problem of increasing the reliability and durability of machine elements and mechanisms in most cases is directly related to the wear resistance of rubbing coupled surfaces. One of the promising ways to increase the wear resistance by obtaining refractory compounds is the method of self-propagating high-temperature synthesis (SHS). The SHS method is based on the exothermic reaction of the interaction of two or more chemical elements, proceeding either in the mode of layer-by-layer directed combustion or in the mode of thermal explosion. Synthesis proceeds in the mode of layer-by-layer combustion when a reaction is initiated in a given section of the sample, and then the formation of a new product proceeds in a narrow zone interspersed throughout the volume of the substance due to heat transfer (Figure 6) [44]. In the thermal explosion mode, the reaction occurs when the entire sample is heated to a given temperature, after which the reaction proceeds almost simultaneously in the entire volume.

The generalized reaction of obtaining a given compound (1) has the following form [30]:

a_i_x_i_ + b_j_y_j_ = Z + Q, (1)

where x—fuel elements (V, Mo, W, Hf, Nb, etc.); y—oxidizing elements (B, C, N, etc.); Z—final product (borides, carbides, etc.); and Q—heat energy of the exothermic reaction.

This technology has found its application in the production of SHS pipes, which are used for the transportation of abrasive materials. They can be used in various industrial applications such as mining, power plants, metallurgy, and construction. The corrosion resistance of these SHS pipes has proven to be much higher than that of steel pipes [44].

#### 2.2.7. Cladding as a Method of Forming Coatings

**Figure 7:**
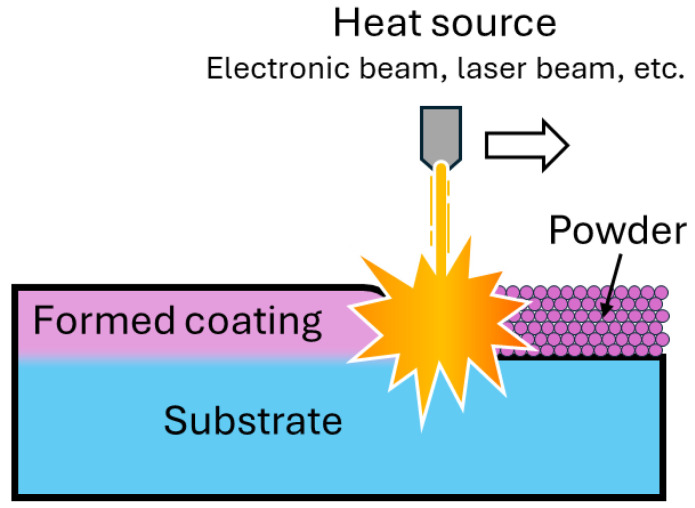
Generalized scheme of cladding processes.

Cladding is a method of depositing a layer of metal or alloy on the surface of an item by means of fusion welding. The melting of the filler layer is carried out by means of an electric or plasma arc, a laser or electron beam, or a high-temperature flame obtained by burning a mixture of combustible gas and oxygen (Figure 7).

One of the most promising methods of applying metallic materials is electron beam cladding. The formation of the coating occurs due to the heating and melting of metal powder of a certain composition previously applied to the surface of the product in a beam of relativistic electrons. The source of electrons is an electron beam gun, which is equipped with a system of electromagnets, allowing a horizontal sweep of the electron beam to be obtained. The formation of coatings can be achieved both in a vacuum and at atmospheric pressure. In the latter case, the power consumption of the unit increases due to electron scattering, but there is a theoretical possibility of coating products of any size. The design of the electron beam gun becomes more complicated. If the thickness of the deposited layer is properly selected, a transition region is formed at the boundary between the coating and the substrate, the composition of which changes in a gradient from the composition of the substrate to the composition of the coating. This provides sufficient adhesion of the cladding to the surface of the workpiece commensurate with the strength of the substrate material. If the thickness of the powder layer is greater than the optimum thickness, only the sintering of the metal powder, which is not bonded to the substrate, occurs. If the thickness of the powder layer is less than the optimum thickness, the surface alloying of the substrate with the powder material occurs. It is also possible to combine electron beam cladding with another coating method. In this case, the coating is applied, for example, by the gas-flame method, and then melted with an electron beam. This eliminates the defects characteristic of gas-flame coatings. Almost any metal alloy can be selected as a coating material. The choice of powder material is limited only by the strength properties of intermetallides formed at the base–coating interface during surfacing [45,46].

The advantages of the method include the high speed of the coating application, the possibility to form coatings of any composition, that coatings obtained by this method have low porosity and good adhesion to the substrate, and that the cladding method can be combined with other coating technologies. The disadvantages of the method include the impossibility of forming coatings on products of complex shape; difficulty in controlling the structure of the obtained coating because the possibilities of controlling the process of crystallization of the melt on the surface of the product are very limited; heating the surface layer of the product during cladding can lead to its deformation; and the high energy consumption [30,45,46].

#### 2.2.8. Electrospark Surface Strengthening (ESA)

Electrospark surface hardening or electrospark alloying is an effective method of surface hardening machine parts made of conventional structural materials. It allows coatings which are firmly bonded to the substrate material and have high-performance characteristics to be obtained [30,47].

**Figure 8:**
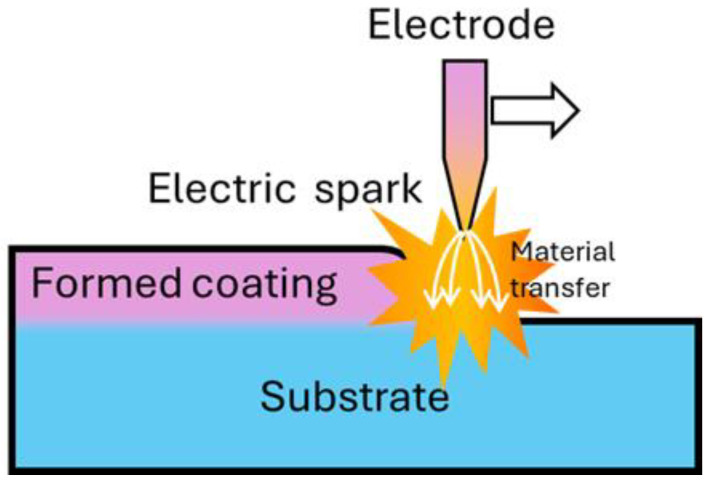
Generalized scheme of ESA.

The ESA process is based on the preferential destruction (erosion) of the anode material by spark discharge and its transfer to the cathode surface (Figure 8).

The main regularities and phenomena determining the ESA processes depend on the material of the alloying electrode and the processing modes. The mechanism of the ESA process is characterized by considerable complexity, representing a combination of erosion, thermal and thermochemical processes, and contact material transfer. The strengthening of the surface layer of the part occurs not only due to the deposition of the anode material but also because of the interaction of this material with the substrate and the formation of solid solutions, chemical compounds, oxides, and nitrides. The hardening effect of ESA increases also because of the pulse effect of temperatures and pressures, leading to structure refinement and the formation of new phases. For hardening electrodes (anode), hard alloys are used, the components of which are carbides of titanium, tungsten, cobalt, chromium, ferrochromium, chromium manganese, and others. The doping of the hardened metal with refractory compounds of boron with titanium, chromium, or tungsten leads not only to the partial dissociation of these compounds in the ESA process and the formation of doped solid solutions but also to hardening by boride particles, which are formed because of the brittle destruction of the electrode (anode) and as erosion products are deposited on the cathode [47].

Electrospark peening does not require preheating and subsequent heat treatment; it does not cause warpage. The hardened layer has high wear resistance and, with sufficient depth and the appropriate selection of electrodes, high heat resistance. The riveting allows the unfavorable influence of electrospark hardening on fatigue resistance to be excluded. The disadvantage of ESA is the complexity of obtaining coatings from non-electrode materials and the need to use compact electrodes. This disadvantage is eliminated when using ESA for coating from powders.

ESA is usually used to increase the wear resistance and surface hardness of machine parts operating in conditions of elevated temperatures and to increase the size of worn machine parts during repair. ESA technology is used for the surface hardening of working parts of road, construction, and earthmoving machines operating in abrasive environments; blades of shot blasting machines; and parts of foundry machinery. Electrospark hardening is also used for the restoration and hardening of landing places in fixed mating and sliding fits. The surfaces of machine parts such as cams, guides, surface locks of keyways, splines, and parts made of structural alloy and carbon steels are subjected to ESA [47].

## 3. Wear and Corrosion Resistance of Refractory Coatings

The comparison of the corrosion protection properties and wear resistance of coatings obtained by fundamentally different methods is a nontrivial and multifactorial task. Wear resistance is determined mainly by the strength properties of the coating, microhardness, strength, adhesion to the substrate, etc., as well as the morphology of the coating and the nature of wear. The corrosion resistance of a coating is determined by many parameters, including the corrosion resistance of the coating material to a given corrosive environment, the coating porosity, the substrate material, the corrosion potentials of the coating and substrate in a given environment, the coating morphology, the nature of corrosion failure of the coating, the homogeneity of the coating material, and so on [48]. The presence of pores in the coating deteriorates its corrosion-protective properties.

The most dangerous types of corrosion for coatings are pitting corrosion and corrosion cracking. Brittle coatings with high internal stresses are most susceptible to corrosion cracking. The propensity to pitting corrosion is determined by the properties of the passive film formed on the coating during exposure to the corrosive environment, as well as by the presence of depassivating agents (usually chloride ions) [48]. Molybdenum, tungsten, chromium, and titanium compounds are considered to be the most resistant against pitting corrosion.

Different methods of hardening hard surfaces lead to ambiguous results. Generally, the wear resistance of a surface increases in proportion to the hardness of the near-surface material. The most wear-resistant coatings can be obtained by gas-phase-diffusion hardening, low-temperature chemical gas-phase deposition, the boriding and chromium plating of steels, and gas–thermal methods. There is a very limited set of methods for producing coatings with a wide range of strength and corrosion-protective properties [1].

### 3.1. Coatings Based on Tungsten, Its Alloys, and Its Compounds

Tungsten coating on copper substrate, which has very important research and application significance, can protect the inner vacuum wall and various internal components from direct irradiation by high-temperature plasma [49,50]. Therefore, various methods of tungsten coating formation on copper substrate are considered: CVD and PVD methods and electrodeposition. The thickness of the formed coatings, depending on the chosen method, varies from 5 to 200 µm. Studies have shown [50,51,52,53,54] that due to the unsatisfactory solubility of W and Cu in each other, it is problematic to organize the “direct” deposition of tungsten coating on copper substrate. As a result, the adhesion resistance of the coating to the substrate is reduced. The coating cracks and peels off. The corrosion resistance of tungsten coatings deposited on copper substrate is high in alkaline environments but low in acidic and saline atmospheres [1,8,55,56]. In [9,51,52,57,58,59], it is shown that in order to combine immiscible metals and thus improve the corrosion and electrochemical behavior of tungsten coatings, electrospark doping can be used, or a suitable interlayer between W and Cu can be created, with promising ones being Ni, Ta, etc.

**Figure 9:**
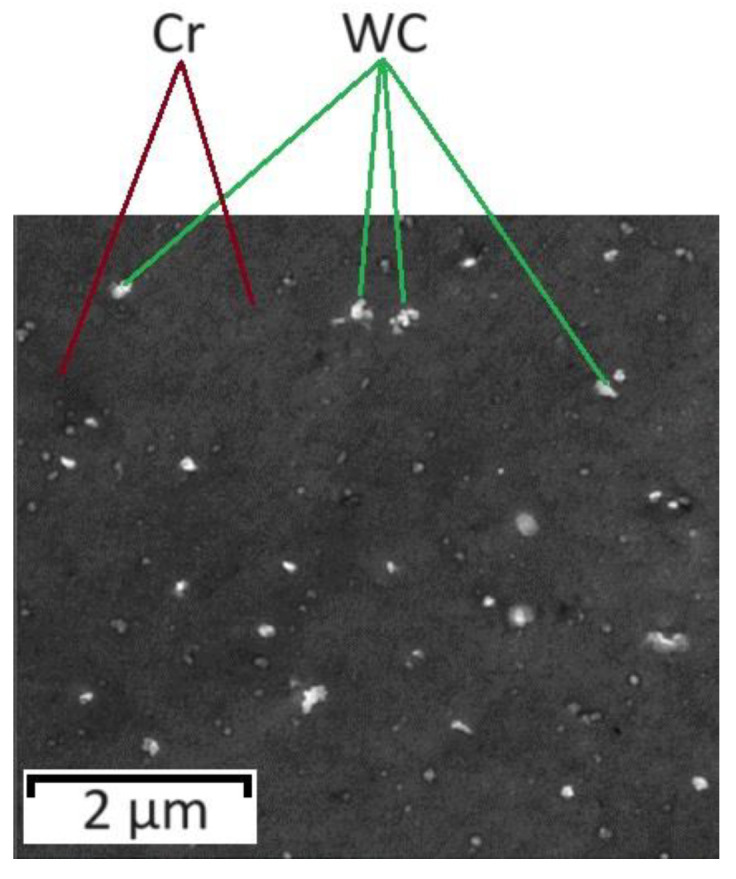
SEM image of composite electrochemical coating Cr–WC [1].

Coatings based on tungsten and its compounds are widely used in industry. Due to their refractory properties, obtaining such coatings by methods based on melting–crystallization (from melts) is technically impossible. In this connection, the technologies of electrochemical and chemical gas-phase deposition as well as the gas–thermal and vacuum spraying of tungsten-containing coatings have become widespread. The problem of obtaining tungsten coatings by the electrochemical method from aqueous solutions is the impossibility of the direct electrochemical reduction of tungstate ions or tungsten oxide with the formation of a metallic phase [1,8,60,61], but it is possible to obtain tungsten alloys with nickel, cobalt, and chromium due to “induced co-deposition” [60,61,62,63,64], which is also relevant for chemical–catalytic metallization processes [57,65]. One of the possible applications of tungsten alloys is their use as promising catalytic active layers for the hydrogen release reaction [66], but there is also a prospect of their use as anticorrosion coatings [58,67]. It has been observed [58] that the introduction of tungsten into nickel alloys leads to an improvement in its anticorrosion properties by reducing the porosity of the deposited layer. An alternative electrochemical method for the preparation of tungsten-containing coatings is the electroplating deposition of CEPs from suspensions prepared from electrolyte solutions and powder materials [1,68,69]. A typical microstructure of ETCs is shown in Figure 9 [1].

Figure 9 shows that carbide inclusions are quite scattered in the chromium structure, and their weight fraction does not exceed 3%. Obtaining a composite electrochemical coating with inclusions of solid particles, as a rule, is aimed at improving the mechanical properties of the coating, and for this purpose, it is more effective to use diamond powders [70], the intrinsic hardness of which is significantly higher than the hardness of tungsten carbide. In terms of increasing the corrosion resistance, it is also worth favoring non-electrically conductive additives, such as Al_2_O_3_, diamond, and SiO_2_, since electrically conductive phases can form a galvanic pair with the base metal, which deteriorates the corrosion resistance of the coating.

Tungsten-containing coatings obtained by gas–thermal spraying have high porosity; therefore, their application as independent anticorrosion coatings without additional treatment—pore filling [32] or thermomechanical treatment [1,8]—is limited. On the other hand, gas–thermal spraying methods allow for the application of composite coatings containing tungsten carbides of unlimited thickness on large-sized products, which allows their application on elements of shut-off valves of pipelines. A promising direction in improving the anticorrosion properties of gas–thermal coatings can be considered to be the alloying of the metal matrix with an easily passivated element [71] or the choice of such an element as a matrix material [72].

PVD coatings typically have a small thickness, and this is due to growth stresses in the coating as it is deposited. These stresses are higher the greater the thickness of the coating. Coatings with a thickness of about 2 µm are considered optimal from a performance perspective [28]. Coatings based on tungsten and its carbides can be obtained by magnetron and vacuum-arc spraying methods [73,74,75], where tungsten obtained by powder metallurgy or chemical gas-phase deposition methods is used as a target material. Data on the corrosion resistance of such coatings are not presented in the literature, but it is known that due to their columnar structure and small thickness PVD coatings have a noticeable permeability [8], which negatively affects their anticorrosion ability.

Coatings based on tungsten and its compounds can be obtained using CVD technology. In this case, the coating is formed from a mixture of gases: tungsten hexafluoride WF_6_, hydrogen H_2_, and propane C_3_H_8_ [76,77,78]. The process allows coatings with a thickness from 0.5 to 500 µm of different phase composition and a microhardness from 4.5 to 40 GPa to be obtained. The regulation of the phase composition of the coating is carried out by means of the ratio of components of the gas mixture WF_6_:H_2_:C_3_H_8_. The microhardness of tungsten obtained by the CVD method is 10 ÷ 30% higher than the microhardness of metallurgical tungsten and is about 5 GPa. Further, when carbon appears in the deposited layers, the microhardness increases up to 17 ÷ 19 GPa. Carbon, in this case, is non-equilibrium-dissolved in the crystal lattice of tungsten, and the excess carbon, interacting with tungsten, is released in the form of W_2_C. The β-W layers exhibit anomalously high hardness, reaching values of 40 GPa, while being a metallic phase. The microhardness of the W-C layers varies within 4.5 ÷ 42 GPa, and no change of precursor or modification of the plant design is required to obtain a coating of a particular hardness.

**Figure 10:**
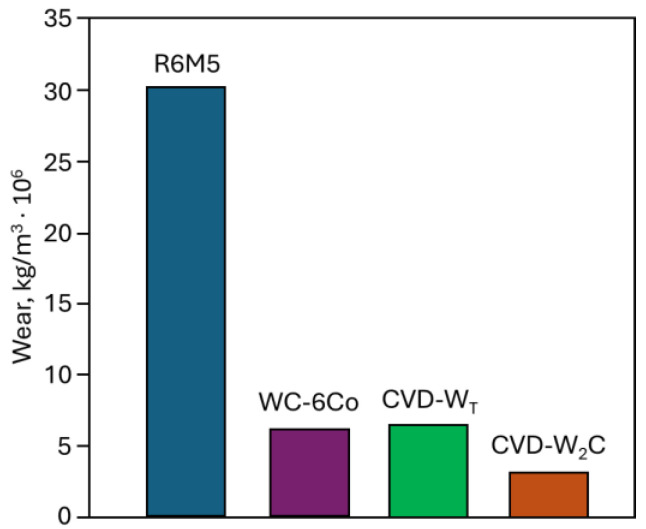
Results of tests for abrasive wear of hard materials and coatings: R6M5—high-speed steel; WC-6Co—cemented carbide; CVD-W_T_—tungsten-based CVD hard nanocomposite, HV = 17 GPa; CVD-W_2_C—CVD tungsten carbide, HV = 33 GPa.

The wear resistance of a hard coating depends largely on its operating conditions. The presence of any liquid or solid particles on the boundary between the rubbing surfaces fundamentally changes the character of their wear. The presence of shock loads can also destroy hard-but-brittle protective coatings, particles of which, getting into the friction zone, aggravate wear. Nevertheless, it is considered that for homogeneous materials with a smooth morphology their wear resistance directly depends on the microhardness. Thus [77], the wear resistance of the W_2_C coating is more than ten times higher than the wear resistance of R6M5 high-speed steel and two times higher than the wear resistance of the WC-Co alloy. The wear resistance of the tungsten-based solid nanocomposite is at the level of tungsten carbide and more than 5 times higher than the wear resistance of high-speed steel R6M5 (Figure 10).

When selecting the coating material, the literature data on the corrosion resistance of tungsten and its carbides obtained by the metallurgical method can be taken into account as a first approximation. However, it should be taken into account that the composition and structural state of the coating material differ significantly from those for metallurgical materials, and the coating thickness is limited to the value of 100 µm for tungsten coatings and 20 µm for carbide coatings. Therefore, to predict the serviceability of the coating, it is advisable to study its corrosion resistance under specific conditions. Thus, tungsten-based coatings are expectedly stable in sulfuric and nitric acid solutions, and tungsten carbide layers are stable in weak nitric acid solutions and in sulfuric acid solutions [8,56,77,78]. In neutral and alkaline solutions of natural aeration, the corrosion rate of tungsten-based coatings does not exceed 20 μm/year [78,79,80,81], but during exposure in a salt spray chamber, the corrosion rate can reach 40 μm/year [8,56], and the first corrosion lesions appear already in the first 20 days of exposure. An important structural feature of CVD coatings is their low porosity, the value of which does not exceed 0.04% for a coating thickness of more than 5 μm [82]. This allows these coatings to be used as independent protective anticorrosion wear-resistant layers that do not require additional processing. However, in the case of the inter-operational storage of products with tungsten coatings, their conservation treatment is required to protect them from atmospheric corrosion. Currently, there are no data on inhibitor surface treatment in the literature, but there is a method of forming protective films from organosilanes, which has shown its effectiveness for protecting tungsten from the effects of neutral salt spray [55,56].

### 3.2. Coatings Based on Tantalum, Hafnium, Vanadium, Their Alloys, and Their Compounds

Of all the currently known metals, tantalum has the highest corrosion resistance, so it is not surprising that tantalum coatings are popular [9,15,16]. Tantalum coatings on copper are applied by PVD (magnetron sputtering, electron beam evaporation), thermal spraying, and electrodeposition. The thickness of the coatings varies from 5 nm to 300 µm depending on the method. Tantalum coatings corrode in the presence of hydrofluoric acid; in a mixture of hydrofluoric and nitric acids; and at room temperature in alkali [9,82,83,84,85,86]. Tantalum-based carbide coatings can be obtained by SHS; plasma spraying; PVD; CVD; and electrodeposition methods [87,88,89,90]. Thus, studies of the corrosion behavior of tantalum carbide coatings on steel substrate in solutions of concentrated inorganic acids (HCl, H_2_SO_4_, HNO_3_, H_3_PO_4_) at room temperature for 30 h showed that the corrosion resistance of products with coatings compared to the resistance of the substrate itself increases by one or two orders of magnitude. Also, the electrochemical behavior of uncoated and tantalum carbide-coated U10 steel was investigated by electrochemical impedance spectroscopy in 3.5% NaCl solution in the frequency range from 10 kHz to 10 mHz and with an amplitude of 40 mV. As a result, the values of the charge transfer resistance R_ct_ for uncoated and TaC-coated U10 steel were found to be 0.82 and 6.62 kOhm/cm^2^, respectively. The capacitance of the double electric layer was also calculated: for steel, it is 6.47 × 10^−5^ F/cm^2^, and for TaC-coated steel, it is 1.20 × 10^−5^ F/cm^2^.

This analysis also confirms that tantalum carbide coating significantly improves the corrosion resistance of steel products [89,90]. The microhardness value of the tantalum-based carbide coating was 34 GPa, which is consistent with the microhardness data of refractory metal carbides. The tribotechnical characteristics of TaC coatings were also investigated. It was found that at room temperature and loads up to 40 MPa the coefficient of friction was within 0.05 ÷ 0.11. No scoring was found on the coating surface after 400 cycles, i.e., TaC coating has high-performance properties [89,90]. Refractory tantalum pentoxide Ta_2_O_5_ coatings are considered as a promising material for use as a wear- and corrosion-resistant material for the protection of components operating under aggressive wear and harsh chemical environments due to its high hardness and exceptional resistance to chemical attack [4,91,92]. Many methods are used to produce tantalum pentoxide coatings, including ALD, magnetron sputtering, etc. [93,94,95]. In [96], the electrochemical behavior of nanostructured Ta_2_O_5_ coating deposited on Ti-6Al-4V alloy was studied. The study was carried out in 3.5% NaCl solution at room temperature, using electrochemical investigation techniques including potentiodynamic polarization, electrochemical impedance spectroscopy, Mott–Schottky analysis, and potential of zero charge (PZC). It was found that the exposure of the coating to chloride-containing solution for 48 h does not significantly change the impedance behavior of the Ta_2_O_5_ coating. At the same time, the impedance values of the Ta_2_O_5_ coating are of the order of 10^7^ Ohm × cm^2^, which is two orders of magnitude higher than that of the uncoated Ti-6Al-4V alloy. The results of the Mott–Schottky analysis and PZC measurements show that the Ta_2_O_5_ coating exhibits a lower carrier density and greater ability to inhibit the adsorption of corrosive chloride ions than the uncoated Ti-6Al-4V. Wear tests show that the presence of a tantalum pentoxide coating on the Ti-6Al-4V alloy results in a decrease of two orders of magnitude in the specific wear rate compared to the uncoated alloy.

The decrease in the wear rate is associated with a decrease in the real contact area and an increase in the surface hardness [97]. In [91], a comparative analysis of the corrosion characteristics of Ta_2_O_5_ and Cr_2_O_3_ nanocoatings deposited on a 100Cr6 steel substrate in 0.2M NaCl (pH 7) at room temperature was carried out. It was found that the steel substrate coated with tantalum pentaoxide has a higher corrosion resistance than the substrate coated with Cr_2_O_3_ [91]. In addition, the method of coating formation also affects the corrosion resistance: Ta_2_O_5_ nanocoatings obtained by the PVD method show almost four times higher corrosion resistance than nanocoatings deposited by ALD. In the process of coating formation by the ALD method, an oxide layer is formed at the coating–substrate interface, leading to the formation of voids at the interface, which contributes to the degradation and dissolution of the coating. In the case of the PVD method, this oxide layer is removed by pre-etching the substrate surface by ion bombardment [94]. As for the tribological characteristics of tantalum pentoxide coatings, the wear resistance of Ta_2_O_5_ nanocoatings increases by two orders of magnitude compared to uncoated 100Cr6 steel. The authors attribute the increase in wear resistance to the good mechanical characteristics of the tantalum pentoxide coating combined with its high adhesion strength [95].

Transition metal nitrides have unique chemical and physical properties: chemical stability, high melting point, high hardness, and high electrical conductivity [98]. Tantalum nitrides are of particular interest for a wide range of technological applications: a stable thin-film resistor for use in silicon-based integrated circuits [99]; a diffusion barrier for bioactive coatings [96,100]; and a material for use in bipolar wafers [99]. Tantalum nitride can be obtained using plasma spraying, SHS, and magnetron sputtering. Thus, in [99], a tantalum nitride nanoceramic coating was applied to bipolar plates of Ti-6Al-4V used in fuel cells using magnetron sputtering to increase corrosion resistance. The microstructure of the coating consisted of small equiaxed Ta_2_N grains with an average grain size of ∼13 nm. The corrosion properties of pure and tantalum nitride-coated Ti-6Al-4V were studied in the simulated environment of the polymer electrolyte membrane of the fuel cell at different pH and temperature values. It was found that the substrate coated with titanium nitride had a higher corrosion resistance than the uncoated substrate at any value of pH and temperature. In addition, the Ta_2_N coating is more hydrophobic than uncoated Ti-6Al-4V, which in turn leads to a reduction in the corrosion behavior of the medium. The corrosion behavior of pure SS 304 stainless steel and that coated with tantalum pentoxide (Ta_2_O_5_), tantalum nitride (Ta_3_N_5_), and tantalum oxynitride (TaON) via magnetron sputtering was investigated in 1 M NaCl solution at room temperature [99]. The formed nanocoatings on the steel substrate showed good local corrosion resistance and improved the protective properties by 50% compared to pure SS 304. The TaON coating showed the best performance, followed by Ta_2_O_5_ and Ta_3_N_5_. This effect is explained by the hydrophobic nature of TaON, which was facilitated by its structure, resulting in a decrease in the corrosion current.

**Figure 11:**
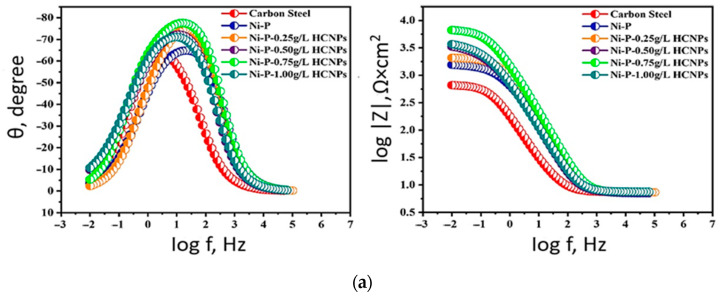

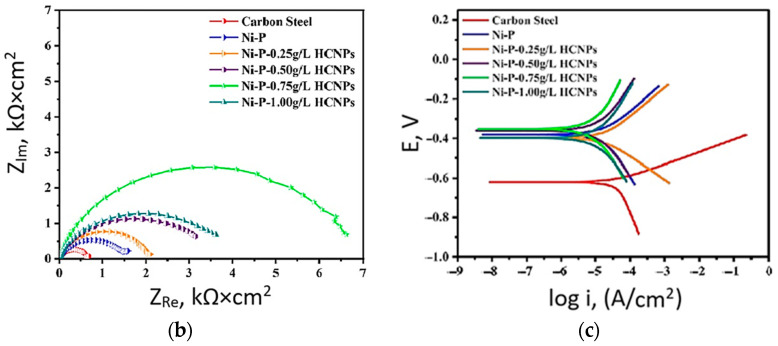
Results of corrosion investigations for pure A36 steel and Ni-P and Ni-P-HfC coatings at C_HfC_ = 0.25, 0.50, 0.75, and 1.0 g/L: (**a**) Bode plot: phase angle variation with frequency and impedance variation with frequency; (**b**) Nyquist plot; and (**c**) Tafel curves [105].

Hafnium is a transition metal with a high melting point, low density, high strength, and better corrosion resistance, as it is practically unaffected by acids and alkalis. Hafnium carbide (HfC) is the most refractory binary compound with the following characteristics: low vapor pressure, a high melting point (3890 °C), high phase stability, and a low oxygen diffusion coefficient [101,102]. The research related to the development of coatings with high mechanical, tribological, and anticorrosion properties led researchers to the creation of composite coatings. Such composite coatings are a binary alloy with solid particles of micro-/nanofillers [103,104,105]. In [105], Ni-P and Ni-P-HfC coatings (HCNPs) were formed on the surface of A36 carbon steel using electrochemical deposition. The concentration of HfC was 0.25, 0.50, 0.75, and 1.00 g/L. The study compared the microstructural, mechanical, and corrosion properties of the formed coatings on the steel surface. In addition, it is necessary to determine the concentration of hafnium carbide leading to the formation of a coating of satisfactory quality. Morphological studies have shown that the Ni-P coating has micropores, while the introduction of hafnium carbide into the system leads to their filling. In addition, increasing the concentration of hafnium carbide leads to an increase in the number of concretions with a decrease in their size, indicating that the HfC particles behave as heterogeneous nucleation sites for nickel and phosphorus ions. This hardening leads to the development of grain growth after deposition on the substrate. The large surface area of the nanoparticles provides grain growth and nodule size. The increase in HfC concentration creates excessive nucleation sites. The nodule size decreases due to the increase in their number. However, when the final value of the nanopores in the cement content is reached, particle agglomeration occurs, negatively affecting the formation of the nanocomposite coating, which becomes a columnar structure with a significant number of micropores (C_HfC_ = 1.0 g/L). Corrosion studies were carried out in 3.5% NaCl solution at room temperature by electrochemical impedance spectroscopy and potentiodynamic polarization, the results of which are presented in Figure 11.

Thus, the study shows that the presence of hafnium carbide in nickel–phosphorus coatings significantly affects the modification of the microstructural properties of the surface. This leads to an increase in the mechanical and anticorrosion properties of HCNP coatings due to the effective blocking of micropores and the dispersion hardening effect. A coating with satisfactory properties was obtained at C_HfC_ = 0.75 g/L. At this concentration of hafnium carbide, the coating shows a high corrosion resistance of 95% and a hardness of 40%, compared to the Ni-P coating. The increase in hardness is attributed to the dispersion hardening effect caused by the presence of hafnium carbide in the Ni-P coating. The obtained coating can be used in the following industries: oil and gas, automotive, aerospace, and many related industries.

Hafnium oxide (HfO_2_) is a material possessing the most important electrical, chemical, and optical properties, such as chemical stability, transparency in the ultraviolet and visible ranges, photoluminescence, a high refractive index and dielectric constant, etc. Due to the described characteristics, HfO_2_ is used in various fields, such as the solar electric power, chemical, photovoltaic, microelectronics, and radio medicine fields [106,107,108,109]. The ALD method is commonly used to form hafnium oxide thin films. In a study [110], the corrosion–electrochemical behavior of ultrathin HfO_2_ films, 15 ± 60 nm thick, formed on the surface of AZ31 magnesium alloy via the ALD method was investigated. Electrochemical studies were carried out in Hank’s balanced salt solution (HBSS), whose pH and ion concentration are close to those of human blood plasma [111]. The corrosion behavior of the films was determined by exposing the samples in a chamber with constant condensation at a temperature of 38 ± 2 °C and a relative humidity of 98 ÷ 100% for 800 h (the time until the appearance of the first corrosion defect was recorded) to determine the corrosion activity at which the practical use of HfO_2_ is possible. It was found that the formed films with a thickness of 15 ÷ 60 nm have an amorphous structure. Depending on the thickness, the color of the films varies: 15 nm—violet; 30 nm—beige; and 60 nm—yellow.

The results of voltammetric studies demonstrated the significant inhibition of magnesium alloy corrosion by amorphous hafnium films. In HBSS solution, the presence of hafnium oxide films on the magnesium alloy substrate led to a decrease in the i_corr_ (A/cm^2^) by at least three orders of magnitude: for pure AZ31, 4.1 × 10^−6^, and for films of different thicknesses 15 ÷ 60 nm, 4.0 × 10^−8^, 5.3 × 10^−9^, and 1.4 × 10^−9^. Such an effect is related to the reduction in the active surface area. The results obtained by electrochemical impedance spectroscopy also confirmed the reduction in the active surface area. Thus, the real part Z_Re_ = R (kΩ × cm^2^) obtained at 0.1 Hz increased by at least two orders of magnitude: for pure AZ31, 5.3, and for films of different thicknesses 15 ÷ 60 nm, to ~200, ~900, and ~4600. The imaginary part Z_Im_ increased from ~0.8 kΩ × cm^2^ for pure AZ31 to ~5000 kΩ × cm^2^ for the sample with a 60 nm thick film. Scanning electron microscopy and energy dispersive spectroscopy revealed pockets of localized corrosion formed under the protective layer. These areas were enriched in oxygen and depleted in magnesium. By means of the exposure of samples with films of different thicknesses of 15 ÷ 60 nm in a constant condensation chamber, the first foci were detected after 160, 240, and 360 h. These test conditions correspond to severe operating conditions. Thus, the obtained films can be used in industrial and coastal areas with moderate salinity, as well as in chemical plants, swimming pools, coastal shipyards for ships and boats, etc.

Vanadium nitrides (VNs) are often used as components for multifunctional coatings that combine the following important properties: hardness, wear resistance, low friction, and corrosion resistance [112,113]. Hafnium nitride (HfN) is a promising and alternative material due to its properties, such as wear resistance, hardness, a high mechanical strength, electrical conductivity, and oxidation resistance under extreme conditions [114,115]. Surface roughness and texture significantly affect the tribological, mechanical, and chemical characteristics of the coating. Oxidation is a key factor in the handling of coated surfaces. Nitrides based on hafnium and vanadium can be obtained by magnetron sputtering. For the considered coatings, not much information on their corrosion properties is available in the current literature. Thus, [115] investigated the electrochemical behavior of HfN and VN layers in 3.5% NaCl deposited on AISI 4140 stainless steel substrate by magnetron sputtering. The thickness of both films was 1.2 µm. The changes in the grain size and surface roughness of the formed films were investigated by atomic force, scanning electron, and transmission microscopy, the use of which revealed that in the case of VN, the grain size was 78 ± 2 nm, and the roughness value was 4.2 ± 0.1 nm; in the case of HfN, the grain size was 58 ± 2 nm, and the roughness was 1.5 ± 0.1 nm. The study of the electrochemical behavior showed that low corrosion rates of 40.870 µm/year for VN and 0.205 µm/year for HfN were recorded for both nitride films, compared to the pure steel surface. The polarization resistance values of the films were also determined, 8.49 kΩ × cm^2^ for VN and 49.34 kΩ × cm^2^ for HfN, which are about 6 and 61 times higher compared to the uncoated steel substrate. This result is attributed to the smaller interplanar distance of HfN compared to VN, grain size, and surface roughness. In any case, the deposition of vanadium and hafnium nitride films by magnetron sputtering on AISI 4140 stainless steel substrate leads to its protection in 3.5% NaCl solution. The corrosion rate for VN is reduced by 95% and that for HfN is reduced by 99% compared to the uncoated substrate.

**Figure 12:**
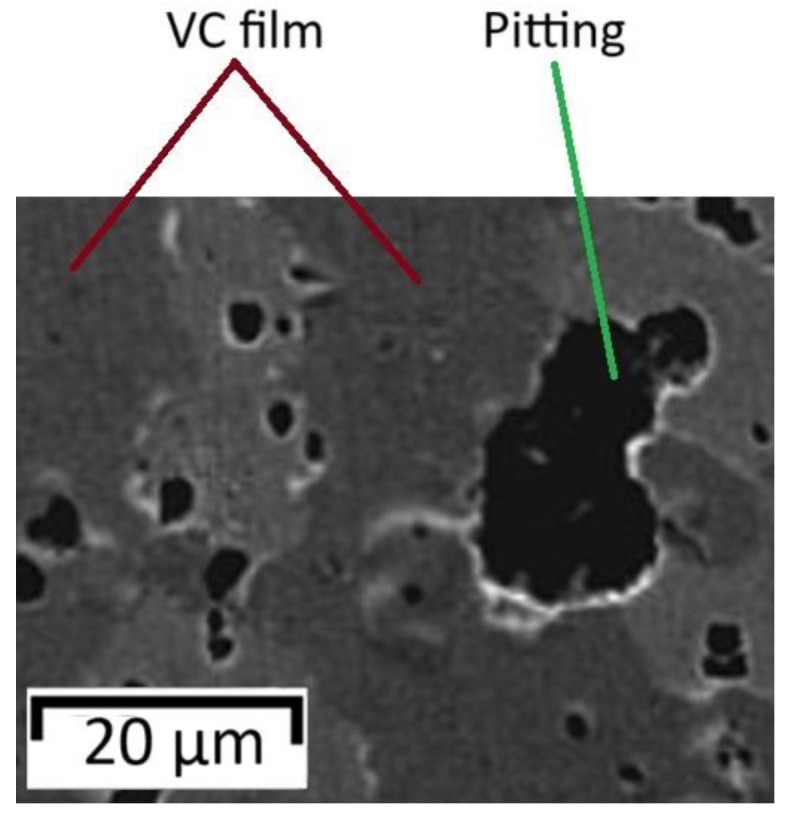
SEM image for the sample C/V = 2.5 after corrosion study [116].

Vanadium carbide is often used for cutting tools due to its good mechanical and tribological characteristics. However, not much information can be found on the corrosion resistance of vanadium carbides. The paper [116] presents data on the electrochemical behavior of vanadium carbide coatings obtained by magnetron sputtering on the substrate of AISI H12 tool steel with direct current at different methane contents and deposition temperatures (from 30 to 400 °C) for the wider use of protective carbon vanadium coatings. The thickness of the formed coatings was constant and amounted to 200 nm. Depending on the change in the methane partial pressure, the ratio of carbon to vanadium content varied (C/V = 0.7, 1.0, 1.6, 2.5). The dependence had a linear character. To investigate the effect of coating composition on pitting corrosion, cyclic polarization tests in 3.5% NaCl were performed for pure AISI H12 and coated steel as a function of the C/V ratio and different coating deposition temperatures: 30, 100, 200, 300, and 400 °C (Table 2 and Figure 12).

It is established that the higher the C/V ratio, the better the protective properties of the coating. In addition, the higher the C/V ratio, the higher the E_pit_ values, i.e., the tendency to pit decreases. The loop closure of E versus j dependence in reverse scanning is also recorded. The values of E_pro_ are more negative than those of E_pit_, which is evidence of repassivation. It is observed that the size of the pitting loop decreased as the C/V ratio increased. The Nyquist diagram shows a depressed charge transfer semicircle at high frequencies for the pure steel electrode, which is attributed to the constant charge transfer time and double-layer capacitance. The intersection of this semicircle with the real axis at high frequencies gives the ohmic resistance of the solution between the working electrode and the reference electrode. At low frequencies, the charge transfer resistance was determined by the difference between the low and high frequencies. For coated electrodes with different C/V ratios, the Nyquist diagrams are similar to those of the uncoated electrode. This indicates that the corrosion failure is controlled by the charge transfer process. Deviations from the ideal circular shape indicate the frequency dispersion of interfacial impedance arising due to the insufficient homogeneity of the electrode surface. The shapes of the curves obtained by electrochemical impedance spectroscopy indicate the presence of open pits, which corresponds to the morphology observed during scanning electron microscopy studies. The impedance value for uncoated steel under these conditions was 0.75 kΩ × cm^2^. For the coated electrodes, the impedance values increased with the increasing C/V ratio, reaching 3.27 kΩ × cm^2^ at C/V = 2.5. Similar trends were also recorded when the deposition temperature was varied at a constant C/V ratio = 2.5, i.e., the corrosion process was attenuated as the coating deposition temperature increased. Such an effect is attributed to the higher concentration of amorphous carbon nanoclusters occupying the voids in the columnar microstructure, which leads to a decrease in the charge flux through the coating. Corrosion can be suppressed by the migration of carbon from tetrahedral sites to octahedral interstitial sites at higher temperatures, which reduces the presence of more reactive carbon in the tetrahedral interstitial positions. Regarding the strength characteristics, it is found that the hardness of the coatings increases sharply when the C/V = 1 ratio is approached and then decreases slowly when the C/V > 1. The Young’s modulus value increases with an increasing C/V ratio and reaches a maximum at a C/V ratio = 1.5. The H^3^/E^2^ ratio also increases with an increasing C/V ratio. The maximum value is recorded at the ratio C/V = 1.2. The obtained data indicate the possibility of optimizing VC coatings in order to simultaneously improve the corrosion resistance and plastic behavior of the coated steel surface by adjusting the deposition temperature, coating composition, and film density.

### 3.3. Iridium-, Ruthenium-, and Rhenium-Based Coatings, Their Alloys, and Their Compounds

**Figure 13:**
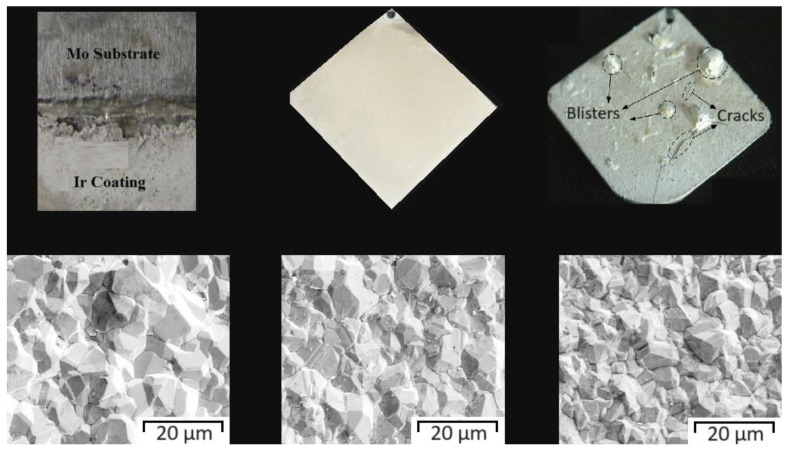
Photographs and SEM images of iridium coatings electrodeposited on substrates: molybdenum, rhenium, and carbon composite [122].

Iridium is a silvery-white refractory metal belonging to the group of heavy platinum metals. Its content in the Earth’s crust is estimated at 10^−7^ %(wt.). In nature, it is found mainly in the minerals nevyanskite, sysertskite, and aurosmiride. Iridium is a metal resistant to aggressive media. At temperatures up to 100 °C, all known acids and their mixtures, including aqua regia, do not interact with it. Iridium can be used in extreme operating conditions not only as an oxidation-resistant material but also as a diffusion barrier for oxygen [13,15]. Iridium coatings can be produced by the following methods: electron beam evaporation, magnetron sputtering, double-glow plasma deposition, organometallic chemical vapor deposition, chemical vapor deposition, pulsed laser deposition, and molten salt electrodeposition [13,15,117,118,119,120,121]. Among these methods, molten salt electrodeposition is ideal for obtaining iridium coatings with high quality and productivity. Thus, [122] conducted a comparative study of iridium coatings obtained by molten salt electrodeposition on molybdenum, rhenium, and carbon composite substrates to investigate the applicability of this method for different refractory substrates. According to Figure 13, it was found that the adhesion strength between iridium coatings and molybdenum and carbon composite substrates is very weak; the coating was removed manually. In addition, cracks and bubbles were observed on the iridium coating deposited on the carbon composite. In the case of the molybdenum substrate, the poor adhesion strength of the coating–substrate is due to the fact that the molybdenum substrate was corroded by dissolved oxygen in the molten salt at the deposition temperature, resulting in the formation of molybdenum oxide. During electrodeposition, needle-like molybdenum oxide is formed at the coating–substrate interface. The liquid molten salt mixture is absorbed by capillary force and remains at the coating–substrate interface during cooling. Thus, inclusions caused by molybdenum corrosion result in the poor adhesion of the iridium coating to the molybdenum substrate. The iridium coating deposited by electrodeposition on the carbon composite substrate is defective and has poor adhesion as a result of thermal stresses occurring when it is cooled to room temperature. This is why the coating develops bubbles, cracks, and peels from the substrate. Applying a transition layer, which will help to relieve the difference in thermal expansion and fill the pores of the carbon substrate, is considered a promising way to solve the above-mentioned problem.

The iridium coating on the rhenium substrate was compact and smooth. The coating–substrate interface exhibited excellent adhesion with no signs of delamination, cracks, or other defects. Neither rhenium corrosion products nor rhenium oxides formed at the interface. This effect is due to the fact that the oxide film formed on the rhenium substrate cannot remain stable in the molten salt at the deposition temperature due to the low melting point of rhenium oxide. The good adhesion strength of the coating to the substrate is attributed to the corrosion characteristic of rhenium in the molten salt and the excellent thermal expansion matching between them.

Besides the fact that iridium can be used for various refractory substrates, its oxides, particularly IrO_2_, are also used to coat electrodes used in the separation of O_2_ and Cl_2_ and in redox coatings, as well as as materials for pH sensors. In a study [123], iridium oxide coatings were obtained by electrodeposition on the surface of stainless steel. The coatings were applied in a standard three-electrode cell using the cyclic voltammetry method. This method was also used to evaluate the effectiveness of the coating applied to stainless steel as a pH electrode sensor. It was found that the thickness of the iridium oxide coating depends on the number of cycles and the scanning speed: it increases with an increasing number of cycles and with decreasing scanning speed. All fabricated pH sensors had a super-Nernstian response in the range of −69.9 ÷ −74.5 mV/per pH unit. Thinner pH sensors showed a faster pH response. The low resistance of the IrO_2_ coating in equivalent circuit models indicates its high electrical conductivity. Three-component anode coatings containing iridium and ruthenium, known as OIRTAs (iridium–iridium ruthenium–titanium oxide anodes) and ORTAs (ruthenium–ruthenium–titanium oxide anodes), are used in industrial chlorine production [124]. In this case, electrolysis is carried out in diaphragm electrolyzers with saturated NaCl solution, and the anode is in an acidic environment, which differs from the conditions of production of low-concentration sodium hypochlorite in diaphragmless apparatuses, where the anode works in an alkaline solution [125]. In practice, hypochlorite can be produced at OIRTAs not only from artificially prepared table salt solutions but also from natural mineralized chloride waters, including sea water. In the study [126], a comparative evaluation of OIRTA and ORTA coatings was carried out during the diaphragmless electrolysis of artificial (3% NaCl) and natural (sea water) raw materials in the production of low-concentration sodium hypochlorite. In the experiments, the ratio of ruthenium to iridium was varied in the range from 0 to 100% with a step of 10%. It was found that the inclusion of iridium in an anodic ruthenium–titanium oxide coating at a percentage–mass ratio of iridium to ruthenium of 80:20 increases the corrosion resistance of anodes. Thus, the average corrosion resistance of the OIRTA was 25 h, which is 8 times higher compared to that of the ORTA coating. Iridium-containing coatings are operated with lower power consumption due to lower voltage at the electrolyzers. The amount of electricity consumed in the production of a kilogram of active chlorine in the optimal mode for a 3% solution is 7.5 kWh/kg; for seawater, it is 10 kWh/kg.

As an improvement in the physicochemical parameters of iridium-based antioxidant protection, a relevant direction is the transition from metallic iridium to alloys and intermetallides based on it. The main attention of researchers is attracted to intermetallic compounds with an L12 structure formed in mixtures of iridium—transition metal of group IV or V [127,128]. These phases have high hardness and strength and among other intermetallides have the greatest thermodynamic stability. Therefore, the intermetallic compound MeIr_3_ is considered an analog of aviation alloys based on nickel [129,130]. The corrosion resistance of intermetallides at high temperatures (2000 ÷ 2500 °C) was investigated in [131]. It was shown that at ~2200 °C the linear recession rate for TaIr_3_ and HfIr_3_ is no more than 25 μm/min in media such as 6.5 F_2_, 10 HF, 6.5 F_2_ + 5.5 O_2_, and 10 HF + 2.3 vol% O_2_. The authors also tested samples based on HfC, TaC carbides, HfB_2_ borides, and HfB_2_–SiC-based compositions. It was found that the linear mass loss rate under similar conditions is over 2 times higher than that for HfIr_3_ and TaIr_3_ intermetallides [131].

The modification of stainless steels with ruthenium makes the resulting alloys competitive with nickel alloys used for processing sulfuric acid solutions at elevated temperatures [132,133,134]. Due to the high aggressiveness of sulfuric acid solutions at elevated temperatures, not many alloys can withstand such operating conditions, which makes it expensive to use alloys with a high nickel content. Sulfuric acid is a by-product of many chemical processes, so the task of finding a corrosion-resistant alloy that could significantly reduce the cost of sulfuric acid treatment is very important. Despite the fact that alloying stainless steels with ruthenium leads to a significant increase in their corrosion resistance in sulfuric acid solutions, the high cost of Ru is a serious problem, which limits the area of its application. An alternative method of improving the corrosion resistance of stainless steels in sulfuric acid solutions at elevated temperatures is the application of thin films on their surface. Ruthenium films can be obtained using the magnetron sputtering method. In [135], the anticorrosion properties of ruthenium films deposited on the surface of stainless steel AISI 304L by magnetron sputtering in a solution of 1M H_2_SO_4_ for 48 h using electrochemical impedance spectroscopy were investigated. It is known that the better the adhesive strength of the film to the substrate, the higher the anticorrosion properties of the film. In this regard, in order to improve the adhesion strength of ruthenium film to stainless steel substrate, the authors tried various methods of preparation of the steel surface before the deposition of the ruthenium film: coarse grinding (substrate roughness 0.83 µm), polishing (substrate roughness 0.05 µm), pulsed deposition (film deposition occurred on a polished substrate under pulsed deposition mode), and an intermediate titanium sublayer (a 2 nm thick Ti sublayer was deposited on a polished substrate). Ru and stainless steel form a non-reactive system; Ti was introduced to improve the wettability of the steel and the adhesion strength of the formed film. The electrochemical impedance spectroscopy results showed a strong dependence of the Nyquist radius on the film preparation method. After 48 h of exposure to 1M H_2_SO_4_, the radii of Nyquist plots increased in the following order: rough grinding < polishing < intermediate titanium sublayer < pulsed deposition. The stability of the films was studied using Bode plots, which showed that in all cases the slopes of the Bode value plots at low frequencies were less than unity, from −0.188 (coarse polishing) to 0.625 (pulsed deposition). This result indicates that the electrode surface is not homogeneous, i.e., the thickness and conductivity of surface oxides changed and discontinuities and porosity occurred in the film. The results of electrochemical studies correlate with the scanning electron microscopy data. The absence of ruthenium film on the sample subjected to rough grinding was recorded. It is possible that the voids formed in the process of grinding were not completely filled with Ru in the process of sputtering. As a result, voids formed at the film–substrate interface, reducing the adhesive strength of the film and increasing its tendency to peel off during operation. The film on the polished substrate showed the presence of wrinkling due to high compressive stresses. In addition, the films on the polished substrate were thicker than the films deposited by the pulse method and Ti sublayer. Thus, the existence of a direct relationship between film thickness and compressive stress in the film is noted: the thicker the film, the higher the compressive stresses in it [136]. The films deposited on the Ti sublayer were featureless; however, defects in the form of blisters were observed on them. The occurrence of blisters, as well as wrinkling, indicates a localized loss of adhesion. Nevertheless, throughout the exposure period, the films performed well, remaining mostly fully adhered to the substrate. For the films obtained by pulsed deposition, localized sites of failure and delamination from the substrate were found. At the same time, they remained mostly intact during the exposure period. The ruptures were observed mainly in the areas of film buckling, suggesting that the fracture is associated with compressive stresses. Importantly, the degree of wrinkling of these films was significantly less when compared to the polished samples. It was necessary to work at a high resolution to capture the defects of these films. This result is consistent with the expectation that if the pulsed deposition method is used, the residual stresses in the ruthenium thin films are reduced, thus reducing the likelihood of wrinkling.

The surface ruthenium alloying of stainless steels gives them high anticorrosion properties similar to bulk alloying. In surface alloying, the amount of ruthenium used is smaller. In this connection, the attention of researchers has turned to nanoscale surface alloys characterized by a small grain size, a large volume fraction of interfaces, and high-anticorrosion properties. Such coatings can be obtained by electrodeposition. In [137], by means of pulsed electrodeposition (PED), the authors formed ruthenium films on the surface of AISI 304L stainless steel and studied their electrochemical behavior in 1M H_2_SO_4_, as well as their mechanical properties. The PED method involves interrupting the coating current for predetermined time intervals called the pulse cutoff time, T_off_. The cutoff time varied from 1 to 45 s. The number of pulses was adjusted so that the total deposition time was 1800 s. It was found that at T_off_ = 1 ÷ 6 s the film cracked and curled, which is associated with high residual stresses. At T_off_ = 12 ÷ 20 s, cracks were recorded in the films, but they were of local character, which is evidence of decreasing residual stresses, and at T_off_ = 30 ÷ 45 s, no cracking and peeling of the film from the substrate was found, i.e., the stress in the film decreased with increasing T_off_. The residual stresses changed from tensile to compressive, and at T_off_ > 20 s, they changed again to tensile. This effect is attributed to the lattice mismatch between the precipitate and substrate as well as hydrogen flow in the surface layer. Electrochemical studies carried out by potentiodynamic polarization showed typical polarization curves characteristic of metals exhibiting active–passive behavior. The value of the corrosion potential for the pure stainless steel surface was −339.8 mV, and for the coated steel at T_off_ = 45 s, it was −200 mV. A similar effect was observed when stainless steel was volumetrically alloyed with 0.2% rhenium [138]. The facilitation of the passivation process is determined by the value of the critical current density, i.e., the lower the critical current density, the easier the passivation proceeds. Hence, increasing the T_off_ favors passivation and improves corrosion resistance. Thus, the R_p_ values at T_off_ = 45 s are four times higher compared to those at T_off_ = 1 s. This result is contrary to expectations, given that R_p_ is inversely proportional to the corrosion rate. Such a contradiction can be explained by considering it in the framework of cathodic modification, which usually increases the corrosion resistance of active–passive alloys. A reduction in the corrosion rate of active–passive alloys can be achieved by introducing metals with high cathodic exchange current density. Such metals increase the kinetic efficiency of the cathodic reaction and shift the corrosion potential towards higher values, at which passivation is facilitated. This is what happened at T_off_ = 45 s. However, the change in the potential alone is not sufficient for spontaneous passivation, as indicated by the values of R_p_. It is likely that the improvement in the corrosion performance with increasing T_off_ is related to the film morphology rather than the amount of ruthenium deposited. The film particles at higher T_off_ were spherical in shape and coarse; this gives greater surface contact for catalytic activity, which is consistent with other data on spontaneous passivation [139]. Importantly, the resulting films remained “stuck” to the stainless steel substrate, which contributed to the observed improvement in the anticorrosion properties of the films.

Another option for depositing ruthenium on the surface of metal alloys is the method of electrospark alloying. In the study [140], the ESA method was used to deposit Ru on the surface of the Fe-40Cr stainless alloy. The authors compared the corrosion–electrochemical behavior of the original Fe-40Cr alloy and the alloy alloyed with Ru using ESA in solutions of 0.5M H_2_SO_4_ and 0.5M HCl at 23 °C. Scanning electron microscopy and energy dispersive spectroscopy results recorded the formation of cellular dendrites in the surface doped layer containing ~52 wt.% Ru. Potentiodynamic studies carried out in 0.5M HCl solution showed that the polarization curve for the initial alloy had a classical active–passive transition. The corrosion potential of Fe-40Cr was −731 mV. In the case of ruthenium alloying, the value of the corrosion potential shifted towards positive values and amounted to −433 mV. In addition, for the Fe-40Cr-Ru sample, no active–passive transition peak was observed on the polarization curve, indicating the onset of spontaneous passivation. A similar type of polarization curve was recorded in the case of studies in a solution of 0.5M H_2_SO_4_. This effect is due to the fact that during the initial selective dissolution, Cg atoms from the alloy escape into the electrolyte. At the same time, Ru atoms diffuse to the formed surface defects of the lattice and accumulate in these places. Under these conditions, the ruthenium content amounted to ~52%. The increase in ruthenium content led to a dramatic increase in the hardness of the laser surface doped layer, with a maximum hardness of 896 HV. The accumulation of Ru atoms on the alloy surface promotes the efficiency of hydrogen evolution. Under these conditions, the corrosion potential shifts to the region of more positive values. Spontaneous passivation will occur when the surface concentration of Ru reaches a critical value. By means of the exposure of Fe-40Cr and Fe-40Cr-Ru samples to solutions of 0.5M H_2_SO_4_ and 0.5M HCl for 11 h after cathodic pre-treatment, it was found that the corrosion potential for the original Fe-40Cr alloy remains in the active region for a long time and is −760 mV in solutions of 0.5M HCl and H_2_SO_4_. In the case of the Fe40Cr-Ru sample, the corrosion potential after cathodic reduction shifts sharply towards positive values and is −356 and −415 mV and then stabilizes to −502 and −445 mV in 0.5M HCl and 0.5M H_2_SO_4_ solutions, respectively. Thus, the deposition of Ru on the surface of Fe-40Cr stainless alloy using the ESA method induces the spontaneous passivation of this alloy under the investigated conditions.

Rhenium is a precious metal whose global production is about five times less than that of platinum. This circumstance makes the use of Re very expensive. In this regard, for the formation of coatings with rhenium in their composition, it is advisable to use rhenium-containing reagents, whose cost is much lower compared to that of pure metal [10,12]. Ni-P coatings formed by electrodeposition are used in the electronics industry, where the quality of interconnections directly depends on the nickel solution and the chemical composition of the coating (phosphorus content). Depending on the phosphorus concentration, the coating can be amorphous or crystalline and contain Ni nanocrystallites. This leads to an increase in its thermal stability temperature, which directly affects the processes occurring at elevated temperatures during soldering or in the further use of the final product (heating/cooling conditions). Modern studies on the reactivity of Ni-P coatings with solders have shown a significant influence of phosphorus on the amount and type of undesirable phases of the Ni_x_P_y_ type occurring [141,142]. Thus, in [143], the reactivity of tin and the corrosion properties of Ni-P and Ni-P-Re coatings obtained on a copper substrate by electrodeposition were investigated. The study of the coating/solder interface showed that the presence of rhenium inhibits the formation of the Ni_2_SnP intermetallic phase, i.e., there is no delamination at the interface. Studies of the corrosion resistance of Ni-P and Ni-P-Re coatings in 0.5M Na_2_SO_4_ solution at room temperature showed a tendency for the corrosion resistance to increase as the pH of the solution from which the coatings were deposited decreased. In 0.5M Na_2_SO_4_ solution for Ni-P coatings, the corrosion resistance increased at the following pH values: 5.6 < 4.8 < 3.8. And in the case of Ni-P-Re coatings, the corrosion resistance increased at the following solution pH values: 5.5 < 4.7 < 3.6. It was found that the addition of rhenium to Ni-P always increased the corrosion resistance of the studied samples.

Rhenium readily forms electrolytic alloys with iron group metals, such as cobalt. The electrodeposition of Co-Re alloy directly depends on the transfer of cobalt released from complex compounds of its ions in the electrolyte solution. In [144], the chemical composition and anticorrosion properties of Co-Re coatings obtained by electrodeposition from citrate and citrate–pyrophosphate electrolyte with pH = 9 for both solutions were compared. It was found that the deposition of alloys from citrate electrolyte can produce X-ray amorphous coatings with a high rhenium content up to 78 at.%. When citrate–pyrophosphate solution is used, cobalt electroreduction proceeds with low overvoltage. In this case, the rhenium content is reduced to 12 at.%, and a coating with a crystalline structure is formed. It was found that there is a relationship between the electrocatalytic and corrosion properties of coatings dependent on the rhenium content in Co-Re alloys. The presence of electrocatalytic properties in coatings promotes hydrogen release in the process of corrosion with hydrogen depolarization, which leads to a decrease in the corrosion resistance of alloys in an alkaline medium. The obtained Co-Re coatings have strong electrocatalytic properties in the hydrogen release reaction. To determine the optimal composition of the electrocatalyst, it is necessary to simultaneously fulfill two conditions: to keep the low overvoltage of hydrogen release and simultaneously the highest exchange current at the lowest overvoltage of hydrogen release. Such conditions are met by alloys with a rhenium content of 60 ÷ 70 at.%. The relationship between the electrocatalytic properties of alloys in the reaction of hydrogen release and the corrosion resistance of coatings in an alkaline medium is fixed for a wide range of rhenium concentrations, for which it has not been traced for electrolytic alloys of other refractory metals, such as Mo and W, due to the impossibility of obtaining alloys containing more than 35 at.% of molybdenum or tungsten.

Inconel 718 (IN718) is a hardened Ni-Cr-Fe alloy with properties such as heat resistance up to 700 °C, high corrosion resistance, and good processability. However, despite such good characteristics, this alloy has unsatisfactory wear resistance. In this regard, IN718 needs additional surface modification, which would improve its tribological characteristics without affecting its anticorrosion properties. In [11], the authors proposed to modify IN718 alloy with rhenium by means of ESA. Two concentrations of rhenium, 14 and 28 wt.%, were used for the ESA process. The formed rhenium coatings had a uniform thickness and a narrow zone without mixing and there was no grain aggregation area. Since rhenium dissolves well in nickel, this allowed for the regulation of the amount of rhenium in the dissolved solid γ-solution by adjusting the laser power and powder feed rate while keeping other process parameters unchanged. The use of the ESA method on the IN718 alloy resulted in the formation of a microstructure consisting of fully (IN718-14Re wt.%) and partially (IN718-28Re wt.%) dissolved rhenium powder in the substrate. During electrochemical studies in 3% NaCl at T = 20 °C, the following was recorded: the value of the corrosion potential for the original sample IN718 was −215 mV, for the electrode IN718-14Re wt.%, E_sogg_ = −114 mV, and for IN718-28Re wt.%, E_sogg_ = −135 mV. The electrochemical behavior for sample IN718 is in agreement with the previously obtained data in [145] under similar conditions. However, the results obtained for samples IN718-14Re wt.% and IN718-28Re wt.% do not correlate with those of [146]. Thus, for sample IN718-14Re wt.%, the value of E_sogg_ increased by 1.9 times while i_corr_ decreased by 1.3 times compared to the original IN718. In the case of IN718-28Re wt., Yesogg increased by 1.6-fold with a 3.6-fold increase in i_corr_. In [147], it was shown that at a rhenium content of 3.5 wt.%, the corrosion potential shifted significantly towards anodic values. At a rhenium content of 6.0 wt.%, the corrosion potential shifted to the opposite region of values. Also, an increase in the passivating current density with increasing rhenium concentration was recorded. This indicates that the passive film appearing on the surface of rhenium layers is less protective compared to the film of the original IN718. In addition, pitting potential data were obtained for the studied samples, which were as follows: for IN718, E_pit_ = 328 mV, for the electrode IN718-14Re wt.%, E_pit_ = 602 mV, and for IN718-28Re wt.%, E_pit_ = 594 mV. The values of potential difference ΔE = E_pi_t − E_sogg_ were as follows: for IN718, ΔE = 543 mV, for the electrode IN718-14Re wt.%, ΔE = 716 mV, and for IN718-28Re wt.%, ΔE = 729 mV. The data obtained indicate an increase in the pitting corrosion resistance [148] with increasing rhenium content. Since the anodic–cathodic transition is more noble than the corrosion potential in the polarization curves, it can be assumed that any film created on the original IN718 surface and in the case of rhenium layers at E_sogg_ may be “not very passivating”, which facilitates the flow of general corrosion. A similar result was obtained in [149]. The alloying of IN718 alloy with rhenium significantly increases the resistance to pitting corrosion but does not increase its passivation ability in chloride-containing solution. To explain the role of rhenium in increasing the resistance to pitting corrosion, it is necessary to take into account the differences in the obtained microstructures. The data of scanning electron microscopy and energy dispersive spectroscopy showed that the corrosion processes are determined by the distribution of segregations of rhenium and niobium. Thus, for the initial sample IN718, irrespective of the development of pitting corrosion, dissolution proceeds mainly in the zones along the grains depleted of Nb and Mo as a result of the deposition of cathode δ-phase and Laves phase on them. In the case of IN718-14Re wt.%, rhenium is concentrated in the center of the γ-phase dendrites, which leads to their anodic dissolution, while the interdendritic spaces with a higher concentration of Nb and Mo, which have a high susceptibility to passivation, are cathodes. For sample IN718-28%Re wt.%, an increased degree of saturation of the γ-phase with rhenium and a high proportion of residual and resolidified Re particles were observed, making the anodic dissolution of these particles the dominant corrosion process. Such corrosion behavior of IN718 alloy with rhenium addition needs further investigation. As for the mechanical performance, it is recorded that in the case of IN718-28Re wt.% the coating is 160% harder, has an 82% lower sliding velocity, and has a 25% higher abrasion resistance compared to the IN718 substrate. Thus, IN718-28Re wt.% is recommended as a wear-resistant coating and IN718-14Re wt.% as a corrosion-resistant coating. It is worth considering the fact that the high cost of rhenium powder can be offset not only by a significant increase in part life but also by reducing laser alloying to small, discrete areas. For example, the consumption of rhenium powder in creating a three-layer coating with a thickness of 6 mm on the sealing surface of a valve of a high-power internal combustion engine is 0.3 g, which is 2.5 and 4 ÷ 5 times lower than that required for manufacturing such a valve from IN718 with 3Re wt.% and 5 ÷ 6 Re wt.% additives, respectively.

Rhenium nitride (ReN) is a superhard and extremely incompressible material. It has a hardness of >40 GPa and a Young’s modulus of 600 GPa. Therefore, it is possible to use this material as an alternative for wear reduction in various industrial applications due to its high hardness and low coefficient of friction [150,151,152]. Today, in the literature, there are data on the formation of ReN coatings by different methods [150,151,152]. The works mainly consider the structural properties of coatings obtained under different conditions. The anticorrosion properties of ReN coatings were not investigated in these works. To improve the anticorrosion properties of coatings, it is possible to apply multilayer systems. Thus, studies of TiAlN coatings have previously shown their high mechanical and protective properties. Thus, it is possible to use TiAlN together with ReN to form multilayer coatings to improve the anticorrosion properties of metal substrates. In [153], a study of the chemical and phase composition and microstructure and anticorrosion properties of multilayer TiAlN/ReN coatings deposited by magnetron sputtering on the surface of steel AISI M2 was carried out. TiAlN/ReN multilayer coatings were formed on the steel surface by monolayer deposition. The total time of the PVD process was 60 min. The varied monolayer deposition times were 2 (C1-ReN sample), 4 (C2-ReN sample), 8 (C3-ReN sample), and 12 (C4-ReN sample) min. The total thickness of the multilayer coating did not exceed 1 µm. The results of phase composition and microstructural analysis showed that in the case of TiAlN/ReN multilayer coatings, rhenium nitride had a cubic crystal structure and also exhibited rhenium precipitates in the form of monoclinic ReO2 oxide and amorphous ReO3 oxide. The multilayer coatings possessed a dense columnar structure with a thickness of 0.87 ÷ 0.97 µm. Electrochemical studies were carried out in 3.5% NaCl for 192 h. The exposure of samples to chloride-containing solution for 192 h showed that the corrosion potential for all multilayer coatings with rhenium content was significantly shifted towards positive values compared to pure AISI M2 steel. The best resistance was demonstrated by the C4-ReN system. Electrochemical impedance spectroscopy results also showed high resistivity values for the coated electrodes compared to AISI M2. Analogously, the resistivity values were significantly higher for the C4-ReN sample compared to other coated electrodes. The potentiodynamic data also confirmed the trend: all coated steel samples showed better protective properties compared to the uncoated steel surface. This effect indicates that all the obtained multilayer coatings containing rhenium are less sensitive to the corrosive environment compared to uncoated steel. Also, during the potentiodynamic tests, the best efficiency was recorded for the C4-ReN sample. The efficiency of C4-ReN in chloride-containing solution under these conditions is explained by a thicker multilayer layer, a higher density of columnar structure, a lower porosity, and the presence of small oxide particles on its surface. According to the obtained results, it can be concluded that multilayer TiAlN/ReN coatings are a barrier protecting the steel substrate from the aggressive effect of the corrosive environment. The penetration of chloride ions is reduced in coatings with thicker monolayers. In [154], an investigation was conducted on the structural features of rhenium nitride deposited on a steel substrate grade H13 by magnetron sputtering, depending on the sputtering conditions. To increase the adhesion strength of the ReN coating to the steel substrate, an intermediate layer of titanium nitride was applied to the steel before sputtering. As a result, it was found that a low nitrogen flow rate and working pressure led to the formation of an unstable coating, i.e., non-stoichiometric ReN with a high content of metallic rhenium was formed. On the other hand, when the nitrogen flow rate and high working pressure were increased, the stabilization of the coatings occurred. As a result, the bonding between rhenium and nitrogen was enhanced, leading to the formation of ReN, and hence, the probability of rhenium oxidation was reduced. The nanohardness value for the stable ReN coating was found to be 12 GPa, which is lower than the theoretical value calculated for this compound. The authors suggest that this effect is due to the lack of stoichiometric coating formation, the coexistence of metallic rhenium and rhenium oxide, and the non-use of bias stress in these coatings.

### 3.4. Niobium-, Chromium-, Molybdenum-Based Coatings, Their Alloys, and Their Compounds

Niobium carbide (NbC) has the following parameters: high hardness, high toughness, high modulus of elasticity, good chemical stability, and wear resistance. Usually, NbC is deposited on materials using the CVD and PVD methods; however, in [155], vacuum condensation reaction sputtering was used to form a corrosion-resistant niobium carbide coating on the surface of AISI 1045 steel. The ferroniobium content of the coatings varied and was 8, 12, 16, and 20 wt.%. Electrochemical studies of the formed coatings and pure steel were carried out in 3% NaCl by means of potentiodynamic measurements and electrochemical impedance spectroscopy. It was found that the thickness of the NbC layers does not depend strongly on the ferroalloy content. As a result, the thicknesses of the NbC layers were 10.88 ÷ 12.95 µm, and their hardness varied in the range of 26.32 ÷ 26.38 GPa. The formed coatings consisted of layers of niobium carbide and niobium oxide (Nb_2_O_5_). These layers were tightly bonded to the metal substrate, indicating that the loss of carbon by diffusion in the substrate does not affect the bonding and cohesive properties of the coatings. Potentiodynamic measurements showed that the presence of niobium carbide on the steel substrate leads to an increase in the corrosion resistance compared to uncoated AISI 1045. Thus, the value of the corrosion potential for the pure steel sample was −831.824 mV. In the case of coated samples, the values of the corrosion potentials shifted to positive values and were approximately the same: −669.470 (AISI 1045/8Nb wt.%), −664.766 (AISI 1045/12Nb wt.%), −672.587 (AISI 1045/16Nb wt.%), and −670.645 (AISI 1045/20Nb wt.%) mV. This effect is due to the formation of Nb_2_O_5_ niobium oxide layers on the coated surface. The amount of Nb_2_O_5_ is constant in each layer. Thus, the presence of this oxide does not affect the corrosion resistance of the coatings depending on the percentage of ferroalloy. It was also observed that the corrosion current slightly decreased with an increasing percentage of ferroniobium. This is probably due to the porosity of the coatings obtained by this method, the presence of which leads to a slight decrease in the corrosion current with an increase in the amount of ferroalloy in the reaction medium. The analysis of corrosion resistance using electrochemical impedance spectroscopy showed that niobium carbide has greater corrosion resistance than uncoated steel only during the first 24 h in a solution of 3% NaCl. The reason for this phenomenon lies in the presence of porosity and defects in the oxide layers of the coating formed at the coating–substrate interface. In [156], the mechanical and electrochemical characteristics of niobium carbide, chromium carbide, and niobium–chromium carbide coatings formed on the AISI D2 surface using vacuum condensation reaction sputtering were compared. The hardness of niobium–chromium carbide was found to be 27.62 GPa, which was higher than the values obtained for niobium carbide, chromium carbide, and uncoated steel coatings: 21.66, 14.71, and 6.70 GPa, respectively. This phenomenon is explained by the presence of two kinds of carbides with different structures and smaller crystallite size in the niobium–chromium carbide coating compared to binary carbides. In addition, the wear resistance of niobium–chromium carbide was also higher compared to binary carbides for uncoated steel. Potentiodynamic measurements in 3% NaCl showed that the best corrosion resistance was exhibited by the niobium–chromium carbide coating compared to other samples. Thus, for pure AISI D2 steel in 3% NaCl, the value of E_sorg_ = −805.5 mV and I_corr_ = 9.72 × 10^−7^ A. Similar data for Nb, Cr, and Nb-Cr carbides are as follows: E_sogg_ = −529.4 mV and I_corr_ = 1.57 × 10^−7^ A, E_sogg_ = −458.6 mV and I_corr_ = 1.22 × 10^−7^ A, and finally, E_sogg_ = −329.4 mV and I_corr_ = 3.17 × 10^−8^ A, respectively. This result is explained by the presence of chromium oxide (Cr_2_O_3_) in the niobium–chromium carbide coating, which forms a protective passive film on the surface of the material. Also, the presence of niobium oxide (Nb_2_O_3_), which has high chemical stability and good corrosion resistance, influences the obtained result [157]. The results of electrochemical impedance spectroscopy in 3% NaCl showed that the corrosion resistance of the niobium–chromium carbide coating is lower than the corrosion resistance of binary coatings. This effect is explained by the smaller crystallite size and porosity of the coating. Thus, the niobium–chromium carbide coatings can be used in applications where good mechanical properties are important but are not suitable for parts exposed to corrosive environments.

Titanium and alloys based on it are biocompatible materials, which makes their use for implants possible. However, due to their high hardness and low thermal conductivity and shape memory, their scope of application is limited [158]. Also, these materials have a high cost compared to other alloys, such as stainless steel. AISI 316L austenitic stainless steel is popular because of its good mechanical properties and high corrosion resistance. These circumstances make it possible to use it in the biomedical field, as well as to use it as an anticorrosive material in aggressive corrosive environments [159,160,161]. AISI 316L is a corrosion-resistant alloy with respect to general and localized corrosion due to the formation of a protective passive film on its surface. Nevertheless, the “failure” of this material due to the development of corrosion processes on its surface remains possible. In this case, the additional protection of the steel surface is necessary to preserve and improve the mechanical, chemical, and biological properties. Thus, the authors of [161] studied the corrosion resistance of niobium and carbon nanofilms applied to the surface of stainless steel grade AISI 316L by magnetron sputtering. Corrosion tests were carried out by exposing pure steel and steel samples with niobium and carbon nanofilms formed on their surface to 0.1M NaCl for 24 hours. Electrochemical studies were carried out in 0.6M NaCl. During tests on all investigated materials, the formation of film destruction was registered. Localized corrosion damage in the form of pits and defects was detected. However, on the sample covered with niobium film, less defects were detected. The data obtained during electrochemical measurements indicate that all formed films on the steel surface work as an effective protective barrier against local corrosion processes. Similar to the corrosion studies, the niobium pentoxide film showed better anticorrosion properties.

In recent years, high-entropy alloys (HEAs) have attracted more and more attention from researchers. The properties of HEAs are usually regulated by bulk alloying; however, this method of adjusting the required properties is expensive. In this connection, it is expedient to use the method of electrospark alloying. HEA FeCoCrNi is increasingly becoming a subject of study due to its combination of good mechanical properties and stability. This alloy has similarities with ductile iron. Ductile iron, due to its high carbon content, is susceptible to the deposition of brittle carbides during the ESA process, which makes it difficult to achieve the desired formability of the coating. In the study [162], coatings based on FeCo0.5CrNi1.5B0.5Nbx, where x = 0.1, 0.2, 0.3, and 0.4, were developed on the substrate of ductile iron QT500-7 by ESA. The effect of niobium content on the evolution of the microstructure and phase composition of the coating was studied to determine the alloy component with the highest wear and corrosion resistance. Corrosion tests were performed in 3.5% NaCl and 0.5M HCl (Table 3).

According to the results presented in Table 3, it can be seen that for the Nb_0.3_ electrode the best result was recorded in both environments: the corrosion potential shifts towards positive values at the lowest corrosion current density. This effect can be explained by the fact that the Nb_0.3_ coating has enough Nb to participate in the oxidation reaction and form a stable passive film [163]. The high carbon content in the coating was attracted by Nb atoms to form a precipitate, which released Cr from the carbonized state to ensure the stability of the passive film. The corrosion resistance of the coatings reached a maximum at Nb_0.3_ and then decreased as the Nb content increased. According to the results of scanning electron microscopy and energy dispersive spectroscopy, excess Nb accelerated the segregation of elements in the alloy, led to lattice distortion, and increased the internal potentiated energy, thereby reducing the corrosion resistance of the coating.

In addition, Nb_0.3_ coatings showed the highest values of hardness (716 HV), H/E, and H^3^/E^2^, as well as a low coefficient of friction of 0.5 and a minimum wear volume of 0.64 × 10^8^, 2.06 × 10^8^, and 5.04 × 10^8^ µm^3^ at loads of 10, 30, and 50 N, respectively. As for the wear mechanism, the following trend was observed for all the studied coatings: adhesive wear was predominant at a 10 N load, oxidative wear was predominant at a 30 N load, and abrasive wear was recorded at a 50 N load.

Chromium coatings are actively used to give surfaces decorative and functional properties [164,165,166]. Due to characteristics such as high resistance to wear, good resistance to corrosion, and high-temperature oxidation, chromium coatings are indispensable in military, aerospace, and other industrial fields [167,168,169]. One of the popular methods for chromium coatings is electrodeposition. Through electrodeposition, it is possible to modify the surface properties and produce metallic materials with desired characteristics. However, in the process of electrodeposition in chromium coatings, it is possible to form texture and residual stresses that negatively affect the operational properties of coatings. In [170], the texture change, hydrogen content, residual stresses, microhardness, and electrochemical behavior of chromium coatings deposited on the surface of stainless steel by electrodeposition were investigated. The coatings on the steel substrate were formed at the following current densities: 20, 35, 40, and 50 A/dm^2^. It was found experimentally that as the current density increased, the average grain size of the coatings changed, which was 0.91, 073, 0.32, and 0.86 µm, respectively. The dislocation density values at the current densities used were as follows: 2.65, 2.24, 2.29, and 1.83 × 10^15^ m^−2^, respectively. A high dislocation density was recorded at 20 and 50 A/dm^2^, and the minimum was recorded at 50 A/dm^2^. It was found that at 20, 35, and 50 A/dm^2^, grain orientation in the (222) plane was preferred, while at 40 A/dm^2^, a mixed orientation in the (222) + (211) planes occurred. It was also recorded that the hydrogen content affects the texture of the coatings. Thus, it was found that a high hydrogen content promotes grain growth along the crystal plane (222). The amount of hydrogen reached its minimum value at 40 A/dm^2^ and amounted to 8.8 ppm. The maximum value of 29.1 ppm was recorded at 50 A/dm^2^. Few microcracks were observed at 35 and 50 A/dm^2^. Low residual stress was obtained at 35 A/dm^2^, and maximum residual stress was recorded at 20 A/dm^2^. As the current density increased in the range of 20 ÷ 40 A/dm^2^, the microhardness increased and amounted to 633, 753, and 820 HV. At 50 A/dm^2^, the microhardness decreased to 702 HV. This result is attributed to grain orientation, grain size, dislocation density, and residual stress. At a current density of 40 A/dm^2^, the mixed orientation of grains in planes (222) + (211) was preferred, and at the same current density, the minimum grain size was recorded. A tendency was found for the microhardness to increase when the residual stress in the coating is high, which agrees with the data [171]. As the dislocation density decreases, the intersection of dislocations during motion also decreases. This leads to a decrease in the accumulation of dislocations, which causes a decrease in the microhardness of the chromium coating [172]. Potentiodynamic measurements were carried out in 3.5% NaCl. The corrosion potential for the uncoated substrate was −513.6 mV, and for the coated samples, it was −410.6, −195.8, −236.4, and −135.4 mV. When the current density was increased in the range of 20 ÷ 50 A/dm^2^, the values of the corrosion current densities were 1.658 × 10^−6^, 0.937 × 10^−6^, 1.315 × 10^−6^, and 0.631 × 10^−7^ A/dm^2^. For pure steel, the corrosion current density was 1.852 × 10^−3^ A/dm^2^. These data indicate that passive films formed on coatings at current densities of 35 and 50 A/dm^2^ have a greater protective capacity than others. The chromium coatings formed at current densities of 35 and 50 A/dm^2^ showed stability, and their corrosion resistance under these conditions was the best compared to other coated samples and clean substrate. These coatings showed the lowest value of current density, which is evidence of the formation of a protective passive film on their surface. The better corrosion resistance of coatings at current densities of 35 and 50 A/dm^2^ is explained by more positive breakdown potential and lower passive current density. The breakdown potential and passive current density at 30 A/dm^2^ were 804.7 mV and 2.220 × 10^−6^ A/dm^2^, and at 50 A/dm^2^, they were 914.8 mV and 1.786 × 10^−6^ A/dm^2^, respectively. Thus, the higher corrosion potential and lower corrosion current density indicate the promising corrosion resistance of coatings formed on steel at 35 and 50 A/dm^2^ [173]. Summarizing the results obtained, it follows that at 35 A/dm^2^ the coating has the least number of microcracks and the best general characteristics. For this coating, higher microhardness, good corrosion resistance, and low residual stress were recorded, which is of great importance in industry for increasing the substrate life and optimizing the chrome plating process.

Another option for the formation of chromium coatings is the ESA method. In [174], the microstructure and corrosion behavior of high-chromium-content stainless steel coatings with a thickness of more than 500 µm deposited on a 27SiMn substrate using different ESA methods, ultra-high-speed (UHS) laser cladding (0.5 m^2^/h) and broad beam laser cladding (BLC), were investigated. The corrosion behavior of the coatings was analyzed in 3.5% NaCl. The solidification of the coatings and their microstructure are influenced by a high cooling rate, when considering the UHS method, and a wide energy input, in the case of BLC. It was found that in the case of UHS, limited energy input occurred, while higher cooling rates led to the formation of a fine and uniform grain structure. The chromium was uniformly distributed in the coating, leading to the formation of an equal-dimensional passive film. The hardness values of the coating formed with UHS were lower (~625 HV) than with BLC (~670 HV), which is due to the higher cooling rate preventing complete martensitic transformation. In the case of BLC, the wide laser beam and slow scanning speed resulted in the formation of larger grains and more complete transformation from austenite to martensite. In this case, chromium was not uniformly distributed across the grains, resulting in non-uniform passive film formation. The corrosion potential, corrosion current density, and pitting potential using the BLC method were −0.35 V, 5.98 × 10^−6^ A/cm^2^, and −0.02 V. In the case of UHS, these values were as follows: −0.25 V, 1.92 × 10^−6^ A/cm^2^, and 0.07 V. The pitting initiated on the BLC coating, in contrast to the UHS coating, occurs at the boundary between coarse and fine grains rather than at defects. The segregation of chromium between grains of different grain sizes and the subsequent inhomogeneity of the Cr_2_O_3_ passive film led to the occurrence of pitting corrosion in the BLC coating. On the other hand, as mentioned above, in the case of UHS, a high cooling rate is used, leading to the formation of an austenitic structure and, as a consequence, a high dislocation density. The presence of dislocations in the coating leads to residual stresses. Since the pittings are stress concentrates, with the imposition of residual stresses, microcracks were found around them, which were not detected in the case of the BLC method. The presence of cracks contributed to the acceleration of corrosion failure. Further development of the BLC method with the purpose of reducing the share of coarse grains and improving the microstructure and composition homogeneity is relevant.

Chromium nitride (CrN) coating is known for its good oxidation resistance, high corrosion resistance, low coefficient of friction, and high wear resistance. In this regard, CrN is widely used in the manufacture of cutting tools, dies, molds, mechanical components, and artificial joints to increase their service life. Recently, more attention has been paid to multilayer nanostructured chromium nitride coatings due to their improved hardness, wear, and corrosion resistance compared to a single-layer CrN coating [175]. Thus, in this work, we studied the corrosion behavior of multilayer coatings applied to the surface of stainless steel AISI 316L using magnetron sputtering in a simulated environment of fuel cells with a proto-exchange membrane (Table 4).

From the data presented in Table 4, we can see that in electrochemical tests all coatings demonstrate good protective properties, but the Cr/CrN coating is the best. The Cr/CrN coating showed the following during potentiodynamic measurements and electrochemical impedance spectroscopy: among all the studied coatings, this coating showed the highest shift in the corrosion potential to the positive region of values, the lowest passive current density, the highest protective effect, and the highest charge transfer resistance, which indicates the lowest corrosion rate in the simulated environment of fuel cells with a proto-exchange membrane. In the study [176], a comparative study of the mechanical and corrosion properties of single-layer CrN and multilayer Cr/Cr_2_N/CrN coatings deposited on an AISI 316L surface by ion sputtering was carried out. The thickness of the multilayer coating was found to be 24.4 µm. The Cr/Cr_2_N/CrN coating exhibited a dense and compact structure. It was also recorded that the adhesion properties at the coating–substrate interface were improved for the multilayer coating compared to the single-layer coating. The hardness value for the multilayer coating was 21 GPa. The multilayer coating reduced the coefficient of friction in both air and seawater compared to the single-layer coating. Potentiodynamic measurements of the coatings in seawater showed that the multilayer coating led to a reduction in the anodic current density compared to the single-layer coating. This effect is due to the structural features of the multilayer coating. Due to the dense structure, fewer defects were formed, and the coating exhibited better protective properties in aggressive corrosive environments. The multilayer structure can limit crack propagation in the layer, which leads to reduced electrolyte access to the substrate and improved anticorrosion properties in seawater.

In recent years, the aerospace and rocket industries have been very active. For these industries, it is important that materials have a high resistance to thermal shock and can also withstand high temperatures. Molybdenum has high thermal conductivity and corrosion resistance as well as high temperature resistance, making it an ideal candidate for use in the aerospace and rocket industries [177]. In [178], the oxidation and corrosion properties of molybdenum coatings formed on the surface of AISI 316L stainless steel using thermal spraying were investigated. The coated samples were subjected to isothermal tests for high-temperature oxidation at 650 °C for 5, 25, and 50 h, as well as isothermal tests for hot corrosion at the same temperature in the presence of 45% Na_2_SO_4_ and 55% molten V_2_O_5_ salts for 1, 3, and 5 h. The volatility of the trio of denominated materials was determined. The volatility of molybdenum trioxide in molybdenum coatings was recorded in hot corrosion and high-temperature oxidation studies. The analysis of the coated samples before and after isothermal high-temperature oxidation tests, conducted using scanning electron microscopy, energy dispersive spectroscopy, and X-ray diffraction, showed that before oxidation tests, the molybdenum coating consisted entirely of cubic Mo phase. After the tests, it was found that, depending on the oxidation time, cubic molybdenum changed to cubic Mo_3_O phase and orthorhombic MoO_2_ phase, which flew away from the structure. Also, under the influence of temperature, changes also occurred in the metallic substrate. The elements Fe and C were transformed into monoclinic compounds Fe_5_C_2_. The remaining Mo, which was not completely removed from the coating structure, reacted with Fe to form the orthorhombic and monoclinic phases Fe_2_(MoO_4_)_3_ and FeMoO_4_, respectively. Usually, monoclinic materials and their alloys have good high-temperature and mechanical (wear-resistant) properties. However, it was found that the resistance of molybdenum to oxidative damage mechanism was weak. Structural transformations had a negative effect on the resistance to hot corrosion. So, after 5 h of corrosion tests in the environment of 45% Na_2_SO_4_ and 55% of molten V_2_O_5_ salts at T = 650 ° C, the molybdenum cover was destroyed. Thus, pure molybdenum coating is not resistant to high-temperature oxidation and hot corrosion. To improve its properties, it is necessary to introduce various alloying elements into the molybdenum coating. After analyzing the literature, the authors suggest that molybdenum-based coatings will perform more successfully when alloying elements and molybdenum itself are not used in pure form. This assumption is a subject for future research.

Niobium-based alloys are widely used as ultra-high-temperature structural materials due to their high melting point, low density, and good mechanical properties under high temperatures [179,180]. However, due to low corrosion resistance to hot corrosion and high-temperature oxidation, the application area of niobium-based alloys is limited. Alloying is an effective way to improve corrosion resistance, but the high content of alloying elements such as Al, Cr, and Si leads to a decrease in their mechanical properties. Thus, in [181], a MoSi_2_/NbSi_2_ composite coating was formed on the surface of a niobium alloy by electrodeposition followed by cementation. The effect of molybdenum on the microstructure of the composite coating and its hot corrosion behavior was investigated. The concentration of ${MoO}_{4}^{2–}$ in the electrolyte was varied: 0.02, 0.03, 0.04, and 0.06 mol/L. Corrosion tests were carried out at 900 °C in the presence of 75% Na_2_SO_4_ and 25% NaCl for 100 h. It was found that the intermediate Ni-Mo layer formed by electrodeposition was denser and showed high adhesion strength to the substrate compared to the electrodeposited molybdenum layer. It was recorded that after grouting, the outer layer of the composite coating mainly contained MoSi_2_, and the sublayer consisted of NbSi_2_. With the increase in Mo content, a decrease in holes and cracks was recorded on the surface of the composite coating. The coating became dense and had a ridge-like morphology. After hot corrosion tests at T = 900 °C for 100 h, it was found that as the Mo content in the composite coating increased, the amount of corrosion products on its surface decreased. Thus, at a ${MoO}_{4}^{2-}$ concentration equal to 0.06 mol/L, the molybdenum content in the composite coating was maximum and amounted to 29.27 wt.%. The parabolic rate constant for the uncoated sample was 3–4 orders of magnitude higher than that for the coated samples. The parabolic velocity of the coated sample containing 29.27 wt.%. molybdenum was the lowest compared to the other coated specimens and amounted to 6 mg/cm^2^. It follows from the results obtained that the MoSi_2_/NbSi_2_ composite coating is able to effectively protect the niobium-based alloy from corrosion under the action of a salt mixture due to the formation of a dense SiO_2_ layer on its surface. Increasing the Mo content in the composite coating leads to an increase in its resistance to hot corrosion.

**Figure 14:**
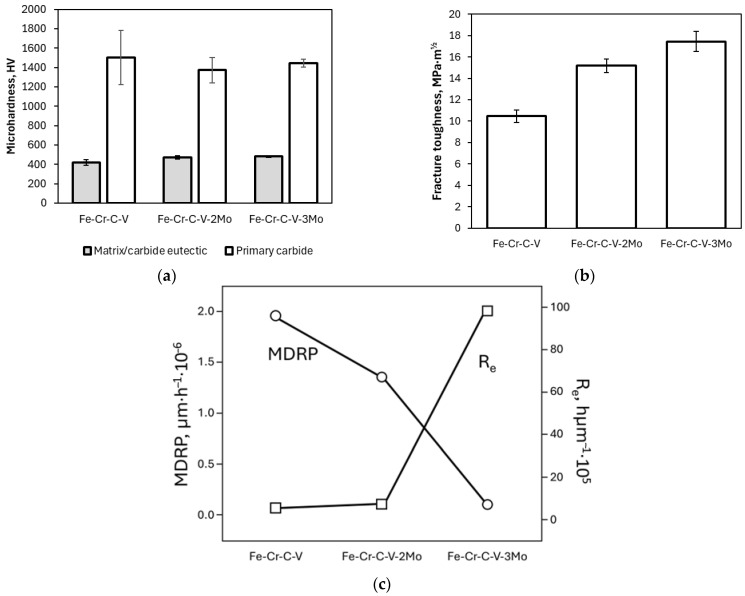
Results of measurements for Fe-Cr-C-V, Fe-Cr-C-V-V-2Mo wt.%, and Fe-Cr-C-V-3Mo wt.% coatings: (**a**) measurements of hardness; (**b**) measurements of viscosity; and (**c**) data on cavitation resistance of the tested coatings [184].

Low-carbon steels are often used for industrial parts due to their high deformation and machinability. However, due to their low hardness, low abrasion resistance, and poor corrosion resistance properties, their application area is limited. To increase the serviceability of parts where high above-mentioned properties are required, alloys based on Fe-Cr-C and Fe-Cr-C-X, where X is carbide-forming elements, except Cr [182,183], are used. In [184], a comprehensive study of the influence of Mo on changes in the microstructure, hardness, wear resistance, cavitation, and corrosion resistance of Fe-Cr-C-V-based hypereutectic cladding alloys deposited on the surface of S235JR steel using electrolight cladding was carried out. The molybdenum content in the coatings was 2 and 3 wt.%. It was found that in addition to the usually present carbides of Cr _23_C_6_, Fe _23_C_6_, Cr_7_C_3_, and Fe_7_C_3_ type in Fe-Cr-C alloys, the presence of molybdenum led to the formation of secondary carbides of Mo_2_C, Fe_2_C, and Cr_2_C type. Also, Mo led to a decrease in the size of primary carbides Cr_23_C_6_, Fe_23_C_6_, Cr_7_C_3_, and Fe_7_C_3_ and their volume fraction. The formation of secondary carbides Mo_2_C, Fe_2_C, and Cr_2_C counterbalances the negative effect of the decrease in the volume fraction of primary carbides, which is necessary to maintain the hardness and wear resistance values at a high level (Figure 14a). Thus, the presence of molybdenum did not change the hardness and wear resistance of the coating (Figure 14 and Table 5). The increased microhardness of the matrix/secondary carbide eutectic as well as the increased indentation toughness resulted in increased cavitation resistance for the Fe-Cr-C-V-V-3Mo wt.% coating (Figure 14b). The maximum cavitation resistance was found for the Fe-Cr-C-V-3Mo wt.% sample, which was 20 times higher than the cavitation resistance of Fe-Cr-C-V (Figure 14c). Table 5 shows that the minimum corrosion rate was recorded for the Fe-Cr-C-V-V-3Mo wt.% coating, which was 4.5 times higher compared to the Fe-Cr-C-V coating. The authors attribute this result to the presence of molybdenum in the matrix and the formation of secondary carbides leading to the formation of a protective oxide layer.

Thus, to increase the cavitation and corrosion resistance without the deterioration of wear resistance, it is recommended to introduce at least 3 wt.% molybdenum into Fe-Cr-C-V alloys.

### 3.5. Osmium-, Rhodium-, and Technetium-Based Coatings

Osmium is a special noble metal used in fuel cell catalysis. It has a high melting point, good thermal conductivity, low compressibility, and a high modulus of elasticity. Various methods are used for the deposition of osmium films: ALD, CVD, PVD, and electrodeposition [14,185,186,187,188]. The use of these methods allows the formation of prepared submicron Os films. However, there is one common problem—the films have low purity, and residual stresses occur in them, leading to their cracking. Thus, the selected deposition method and its parameters determine the quality of the osmium film.

Acidic and alkaline electrolytes are used for osmium electrodeposition. An electrolyte based on hexachlorosmiate was patented, from which osmium coatings up to 1µm thick were formed. Thin coatings with high internal stresses were deposited from a closely related electrolyte. At a thickness of 1.5 µm, they were covered with a crack network. An acidic solution of sodium hexachlorosmiate produced dark precipitates weakly bound to the substrate. Compact osmium coatings 2 ÷ 3 µm thick were obtained from electrolytes prepared by dissolving osmium nitrosohydroxonitrite in sulfamic or hydrochloric acids. However, these electrolytes were unstable and could not be recommended for industrial use. The coatings at a thickness of 1 µm were covered with a network of cracks. Significantly better results were obtained when osmium was precipitated from alkaline electrolytes. Thus, from the boiled electrolyte of osmium oxide (VIII) with sulfamic acid, pH 14, a shiny osmium coating was obtained, firmly bonded to the substrate with a thickness of 1.3 µm. At thicknesses greater than 2.5 µm, dull coatings with cracks were obtained. The wear resistance of the obtained osmium coatings exceeded the wear resistance of the rhodium and chromium coatings. An alkaline electrolyte containing 8 ÷ 10 g/L osmium (in terms of metal) and 50 ÷ 56 g/L potassium hydroxide (total) was obtained by the anodic dissolution of osmium with separated anodic and cathodic spaces. At a cathodic current density of 20 ÷ 25 A/dm^2^ and temperature of 35 ÷ 40 °C, it was possible to form a compact osmium coating on copper substrate with a thickness of 1.0 ÷ 1.2 µm, which was porous already at a thickness of more than 0.6 µm. Comparative tests of osmium (obtained in alkaline electrolyte), palladium, and rhodium coatings applied to copper showed that at all contact loads the transient resistance of osmium coatings was the lowest, and the wear resistance (tests on friction mock-up, counterbody—leather) and corrosion resistance (in SO_2_ atmosphere, in salt fog chamber and humidity chamber) were the highest. Osmium coatings formed from an acidic electrolyte were characterized by high porosity and were not subjected to comparative tests [13]. The use of magnetron sputtering allowed the deposition of the osmium film on the quartz surface. To improve the adhesion strength of the film, an intermediate titanium layer ~100 nm thick was formed on the quartz surface prior to deposition. As a result, the formed osmium film had high purity, good adhesion to the substrate, and a thickness of ~3 µm. The hardness of the film was 40% higher than that of the solid sample due to minimal internal stresses (~5%) and crushed grains (~33%). The dependence of the resistivity of the film on its thickness was established: ρ = 13 + 1.74/t (μΩ × cm), where t is the film thickness in µm.

The most used method of rhodium plating is electrodeposition, thanks to which the application area of Rh plating is expanding, despite the high cost of this metal. This is due to the high corrosion resistance of rhodium coatings, high long-term light reflection coefficient, low contact resistance, high electrical conductivity, high hardness, wear resistance, and beautiful appearance. Unlike silver, rhodium has a long-lasting ability to reflect light rays. Therefore, rhodium electrochemical coatings are used to protect the surface of silver mirrors and reflectors from tarnishing [13,23]. Rhodium is highly resistant to hydrogen sulfide and sulfur compounds. It is resistant to all alkalis, inorganic and organic acids, and even to aqua regia. Due to their high corrosion resistance, rhodium coatings retain good conductivity in contacts for a long time, which in combination with their high hardness and wear resistance determined their use for coating precision conductive, sliding, and rubbing contacts of radio and electronic equipment, requiring trouble-free operation in difficult conditions.

Rhodium coatings are used as a barrier layer between gold and copper and silver and nickel–iron alloy to prevent mutual diffusion. Rh coatings are also used along with platinum coatings in the jewelry industry. While not inferior to the latter in appearance and resistance, they are more favorable due to their much lower density. To apply a layer of the same thickness, 42% less rhodium is used than platinum. Usually, thin layers of rhodium are applied for protective and decorative purposes: to protect silver from tarnishing, 0.13 ÷ 0.25 µm; to cover the surface of reflectors, 0.25 ÷ 0.40 µm; for lightly loaded moving electrical contacts, 0.3 ÷ 0.5 µm; for loaded contacts, 4 ÷ 5 µm; and for contacts subjected to strong mechanical impact, up to 10 ÷ 20 µm. Rhodium coatings are characterized by high internal stresses (200 ÷ 2000 MPa), the value of which depends on the composition of electrolytes, the methods of their preparation, and the mode of electrodeposition. A sharp increase in internal stresses, accompanied by the cracking and peeling of coatings, causes contamination by organic compounds and salts of heavy metals, to which rhodium electrolytes are very sensitive. Rhodium can be directly deposited on silver, gold, copper and copper alloys, and nickel and nickel alloys. When deposited on beryllium bronze, a layer of copper is applied beforehand. When depositing rhodium on tin zinc alloys, aluminum is applied with a sublayer of nickel or silver. Sulfate–sulfamate and amino chloride electrolytes are used for the electrodeposition of rhodium. The literature mentions electrolytes based on rhodium chloride, aminonitrite and diaminonitrite rhodium co-compounds, and rhodium fluoroborate. The most widely used are sulfate electrolytes [13].

Technetium (Tc) is the first radioelement of the Periodic Table. D. Mendeleyev predicted its existence back in 1869; however, Tc was discovered only in 1937 by C. Perrier and E. Segre. Tc has two isotopes: ^99^Tc (2.1 × 10^5^ y), a by-product of the nuclear industry, and ^99m^Tc (6 h), used in medicine as an imaging agent [189]. The Tc content in the Earth is not particularly high. This element is present in the environment as a product of the spontaneous fission of uranium. Technetium is mostly obtained from spent nuclear fuel or by neutron irradiation. Although Tc was discovered more than 85 years ago, there are not many laboratories that can afford to conduct research with this expensive radioactive element. In this regard, the chemical behavior of Tc is not as well developed as that of its Group 7 members, such as Mn and Re. Research related to applied technetium electrochemistry has focused on the development of sensors for the quantitative detection of ^99^Tc in the environment and industrial samples. Such sensors can be used to study remediation activities at nuclear facilities. Electrochemical studies have shown that ^99m^Tc can be separated from Mo targets. The task of developing efficient electrochemical methods for separating ^99m^Tc from ^235^U remains relevant. Future studies should focus on understanding the redox properties of Tc. This research should lead to a new way to produce metallic Tc and alloys, as well as new separation methods that could be used in the nuclear or radiopharmaceutical industries [22].

## 4. Comparison of the Characteristics of the Considered Coatings Based on Refractory Materials

The unique physical properties of refractory materials, including their high melting point, sharply limit the range of methods for obtaining coatings based on them. However, the methods discussed in this article are based on the application of high energies or chemical and electrochemical processes, which not only allows the deposition of refractory compounds in the form of coatings but also leads to the formation of special structural states in them, as well as the achievement of high protective properties. Table 6 summarizes the refractory coatings considered in this review.

Thus, the PVD and CVD methods are mostly capable of producing refractory coatings. Some refractory metals such as Nb, Cr, Ir, and Os can be obtained by electrochemical reduction from electrolyte solutions or melts. Thermal spraying allows for the deposition of composite coatings that include refractory carbides and nitrides. The structure of a coating is determined mainly by the method of its production. PVD and CVD allow nanostructured coatings to be obtained.

## 5. Conclusions

This paper presents a comprehensive review of micro- and nanostructured coatings based on refractory metals. This review considers various methods of synthesizing refractory coatings and also presents information on their anticorrosion and physical and mechanical properties. From the reviewed data, it follows that it is impossible to say unequivocally that the nanostructure contributes to the enhancement of anticorrosion and physical–mechanical characteristics in comparison with microstructural coatings. In pursuit of increasing the anticorrosion properties, the physical and mechanical characteristics of refractory coatings are found to suffer and vice versa. Thus, when creating a coating based on refractory materials, it is necessary to clearly understand what “strong” properties (anticorrosive, physical–mechanical, tribological) are necessary under the given operating conditions. Depending on the purpose, the most suitable synthesis method can be chosen, leading to the formation of a high-quality and inexpensive method of synthesis of refractory coating. It is also worth considering that the use of some methods, in particular, chemical heat treatment, electrospark alloying, and cladding, allows the use of these metals as alloying additives in the composition of the surface layer, which is a saving approach to improving the anticorrosive, physical–mechanical, and tribological characteristics of the protected material.

## Figures and Tables

**Table 1:** Brief data on melting points and applications of refractory metals.

Metal	Melting Temperature, °C	Application	References
W	3400	Material of electrovacuum devices; the main component for the manufacture of filaments of incandescent lamps, as well as high-voltage cathodes of high-power generator lamps and X-ray tubes with operating temperature 2200 ÷ 2800 °C.	[1,8,9]
Re	3180	It is used for electrical contacts operating in conditions of high temperatures and humidity and in the electrovacuum industry for filaments of electric lamps, cathodes, grids of radio lamps, material for heat-resistant alloys, electroplating, springs, and other parts, in particular for wear parts.	[10,11,12]
Os	3033	Material for production of catalysts for ammonia synthesis, hydrogenation of organic compounds, support axes of precision instruments, and pen tips for fountain pens. A component of superhard and wear-resistant alloys with iridium and ruthenium; in combination with tungsten, it is used for filaments of incandescent lamps. It is used in jewelry. Osmium tetraoxide is used in electron microscopy to fix biological objects.	[13,14]
Ta	3000	Material for the production of shaped parts, wire, and foil, thickness up to 10 µm; used in the production of electrolytic and thin-film capacitors of high specific capacitance, obtained by anodic oxidation, as well as in electrovacuum technology for critical parts: anodes and grids of generator lamps and incandescent cathodes.	[9,15,16]
Mo	2620	It is used for manufacturing parts of complex configuration, as a material for heating elements in high-temperature electric furnaces (up to 1700 °C), and as a material for grids and electronic lamps and other electrovacuum devices (hooks, threads, suspensions).	[17,18]
Nb	2500	Material for the manufacture of capacitors used in radio electronics and microprocessor technology; it is suitable for the manufacture of containers for transportation and storage of radioactive waste due to low radioactivity and is also an alloying additive in the production of steels.	[16,18]
Ir	2446	The material is used for production of spark plugs in internal combustion engines, production of heat-resistant crucibles used for growing single crystals of precious stones, creation of catalysts used in the chemical industry, production of mouthpieces through which refractory glass is blown, devices for electrical stimulators of cardiac activity, and creation of pieces of jewelry, which are distinguished not only by their decorative properties but also by their durability.	[13,15]
Ru	2334	It is used as a material for the manufacture of research and therapeutic drugs, for the manufacture of catalysts used in water purification spacecraft, and for the manufacture of turbine blades of jet engines, high-temperature parts of rockets, and hardware of lethal devices. It is also used in the electronics industry and as an alloying additive in metallurgy.	[19,20]
Hf	2225	It is used in the production of special grades of glass for fiber-optic products, as well as for the production of high-quality optical products and coating of mirrors, including night vision devices and thermal imaging cameras.	[15,21]
Tc	2157	Widely used in nuclear medicine for the brain, heart, lungs, etc., as well as for diagnostics of tumors in computer tomography. Material for matrix of radioactive waste, for obtaining ruthenium during transmutation in nuclear reactor, and for anticorrosion protection of cooling circuits of nuclear reactors.	[22]
Rh	1963	It is used for production of catalysts for exhaust system units, in durable heat-resistant chemical ware, in thermoelectric measuring equipment, for manufacture of detectors for nuclear reactors, for improving decorative properties of gemstones, in devices, and as a chemical element for nitric acid and other reactions. In electrical engineering, it is used as a material for covering connectors, contacts, and reflectors.	[13]
Cr	1900	It is used for protective coatings of products (chrome plating), including those operated at elevated temperatures. It has good adhesion ability to glass, ceramics, and sitall and is well compatible with other conductive materials, so it is used in microelectronics for the manufacture of resistors, adhesion sublayers for contact pads, and conductive connections.	[23,24]
V	1887	It is used in the nuclear power industry as a material for fuel element cladding and tubes and in the production of electronic devices, as well as as an alloying additive in the production of high-strength low-alloy steels.	[16,25]

**Table 2:** Relevant potentials of E_pit_ and E_pro_ for pure AISI H12 and coated steel depending on the C/V ratio of the coating in 3.5% NaCl solution.

Sample	E_pit_ (mV)	E_pro_ (mV)
AISI H12	−496	−663
C/V = 0.7	−487	−673
C/V = 1.0	−444	−688
C/V = 1.6	−417	−662
C/V = 2.5	−414	−649

**Table 3:** Polarization curve fitting parameters for pure QT500-7 and HPP FeCo0.5CrNi1.5B0.5Nbx, where x = 0.1, 0.2, 0.3, and 0.4 in solutions of 3.5% NaCl and 0.5M HCl.

Media	Parameter	QT500-7	Nb_0.1_	Nb_0.2_	Nb_0.3_	Nb_0.4_
3.5%NaCl	E_corr_, mV	−856	−388	−459	−395	−461
	i_corr_ × 10^−6^, A × cm^−2^	8.19	2.10	1.25	0.21	1.41
0.5M HCl	E_corr_, mV	−451	−271	−273	−337	−355
	i_corr_ × 10^−6^, A × cm^−2^	8.75	2.64	1.50	1.10	1.45

**Table 4:** Results of potentiodynamic measurements for stainless steel and various multilayer nitride coatings in 1M H_2_SO_4_ + 2 ppm F at 70 °C for 168 h.

Sample	E_corr_, mV	i_corr_, µA/cm^2^	Protective Efficiency, %	R_ct_, MΩ × cm^2^
Substrate	−300.4	115.650	–	1.7
Ti/TiN	−5.6	1.312	98.87	3.2
Cr/TiN	12.0	1.201	98.96	4.6
Ti/CrN	213.0	0.273	99.76	4.8
Cr/CrN	447.8	0.003	99.99	10.0

**Table 5:** The results of corrosion tests for Fe-Cr-C-V, Fe-Cr-C-V-V-2Mo wt.%, and Fe-Cr-C-V-3Mo wt.% coatings in 0.4M NaCl.

Sample	E_corr_, V	E_pit_, V	i_corr_, µA	R_p_, kΩ	Corrosion Rate, mm/Year
Fe-Cr-C-V	−0.303	−0.230	2.591	17.670	0.90
Fe-Cr-C-V-2Mo wt.%	−0.295	−0.005	1.211	28.515	0.34
Fe-Cr-C-V-3Mo wt.%	−0.316	−0.025	0.835	33.025	0.20

**Table 6:** Methods of production and features of coatings based on refractory materials.

Materials	Method of Coating Production	Features
W, WC, and W_2_C	CVD, PVD, thermal spray, and electrochemical deposition (as part of composite coating)	Using these methods, coatings based on tungsten and its compounds with high wear resistance and protective properties compared to the substrate are formed. This allows these coatings to be used as independent protective anticorrosion wear-resistant layers that do not require additional processing. However, in the case of the inter-operational storage of products with tungsten coatings, their conservation treatment is required to protect them from atmospheric corrosion.
Ta, TaC, Ta_2_O_5,_ Ta_2_N, Ta_3_N_5_, TaON, Hf, HfC, HfO_2_, HfN, V, VC, and VN	PVD (magnetron sputtering, electron beam evaporation), CVD, SHS, ALD, thermal spray, electrochemical deposition, and plasma spray	The use of these methods leads to the formation of nano- and microstructural coatings of tantalum, hafnium, vanadium, and their compounds on a metal substrate. In comparison with the metal substrate, a “strengthening” of anticorrosion and mechanical properties is observed.
Ir, IrO_2,_ Ru, Re, and ReN	CVD, PVD (magnetron sputtering, electron beam evaporation), double-glow plasma deposition, pulsed laser deposition, and electrodeposition from molten salts	These methods lead to the formation of microstructured coatings of iridium, ruthenium, rhenium, and their compounds on the substrate surface. The anticorrosion and mechanical properties of the coatings are significantly increased compared to the substrate; in particular, the resistance to localized corrosion is improved. However, the obtained values of mechanical characteristics of coatings are lower than the calculated values, which is due to the non-stoichiometricity of the compounds in the composition of these coatings.
Nb, NbC, Nb_2_O_5,_ Cr, CrC, Cr_2_O_3_, CrN, Mo, and MoSi_2_/NbSi_2_	CVD, ESA, magnetron sputtering, vacuum condensation spraying, electrochemical deposition, and thermal spraying	The formation of both nano- and microstructured coatings of niobium, molybdenum, chromium, and their compounds on metal substrates has been recorded as a result of these methods. Niobium–chromium carbide coatings can be used in applications where good mechanical properties are important but are not suitable for parts exposed to corrosive environments. Molybdenum coating is not resistant to high-temperature oxidation and hot corrosion. To improve its properties, various alloying elements need to be introduced into the molybdenum coating. Composite MoSi_2_/NbSi_2_ coating protects the metal substrate from corrosion under the action of salt mixture due to the formation of a dense layer of SiO_2_ on its surface.
Os and Rh	ALD, CVD, magnetron sputtering, and electrochemical deposition	The application of these methods of coating formation leads to the formation of micron-thick films of osmium and rhodium on the substrate, which have good anticorrosion and mechanical characteristics.

## Data Availability

Not applicable.
